# Supplementary material for: Structure-guided insights into heterocyclic ring-cleavage catalysis of the non-heme Fe (II) dioxygenase NicX
Source: Nat Commun. 2021 Feb 26;12:1301. doi: 10.1038/s41467-021-21567-9 (PMC7910607; doi:10.1038/s41467-021-21567-9)
Supplement: Supplementary file 1 — Supplementary Information [file 41467_2021_21567_MOESM1_ESM.pdf]

Supplementary Information

Structure-guided insights into heterocyclic ring-cleavage catalysis of the non-heme Fe (II) dioxygenase NicX

Gongquan Liu[1†], Yi-Lei Zhao[1†], Fangyuan He[2], Peng Zhang[2], Xingyu Ouyang[1], Hongzhi Tang[1*], and Ping Xu[1]

[1]State Key Laboratory of Microbial Metabolism, Joint International Research Laboratory of Metabolic and Developmental Sciences, and School of Life Sciences and Biotechnology, Shanghai Jiao Tong University, Shanghai, People's Republic of China

[2]National Key Laboratory of Plant Molecular Genetics, CAS Center for Excellence in Molecular Plant Sciences, Institute of Plant Physiology and Ecology, Shanghai Institutes for Biological Sciences, Chinese Academy of Sciences, Shanghai, People's Republic of China

†These authors contributed equally to this study.

*Corresponding author: H. Z. Tang

Mailing address: School of Life Sciences & Biotechnology, Shanghai Jiao Tong University, Shanghai 200240, P. R. China

Email: tanghongzhi@sjtu.edu.cn; Tel: +86-21-34204066; Fax: +86-21-3420672

Supplementary Table 1. Kinetic analysis of NicX wild type and mutants.

Enzymes	K_m (μM)	V_{max} (n kat mg^{-1})
Wild type	94.9 ± 3.84	58.62 ± 0.95
C76A	103.4 ± 3.33	8.00 ± 0.11
C76Q	N.D.[a]	N.D.
C76E	N.D.	N.D.
L104A	112.3 ± 4.03	2.07 ± 0.03
H105A	N.D.	N.D.
H105F	142.1 ± 8.63	4.12 ± 0.10
H105M	145.5 ± 2.41	6.33 ± 0.04
V175F	117.9 ± 3.68	13.08 ± 0.18
E177A	260.1 ± 10.85	3.96 ± 0.07
H189A	211.9 ± 6.60	1.59 ± 0.02
E177A/H189	N.D.	N.D.
H265A	N.D.	N.D.
R293A	N.D.	N.D.
S302A	N.D.	N.D.
E308A	95.85 ± 3.68	26.12 ± 0.20
H318A	N.D.	N.D.
D320A	N.D.	N.D.

a.N.D. denotes that the kinetic constants were beneath the measurable limits of detection, and were therefore not detectable.

Supplementary Table 2. ICP-MS analyze the Fe(II)-binding capacities of NicX-WT and its mutants.

Enzymes	Concentration of protein (μM)	Concentration of Fe (μM)
Wild type	8.5	7.21
H265A	8.4	0.542
S302A	8.2	0.446
H318A	7.6	0.328
D320A	8.0	0.340
R293A	8.8	0.416

Supplementary Table 3. Secondary structure analysis of NicX and its mutants based on circular dichroism spectra.

Enzymes	α-helix	β-sheet	Random
WT	9.60%	57.00%	33.40%
H265A	10.20%	57.00%	32.80%
R293A	10.60%	57.20%	32.20%
S302A	10.40%	57.20%	32.40%
H318A	10.30%	57.50%	32.20%
D320A	9.80%	57.30%	32.90%

Supplementary Table 4. Primers used in this study.

Primer	Sequence
nicX-WT-f	5’-agtcatatgccggtgagcaatgcacaa-3’
nicX-WT-r	5’-tataagctttcgcgctcgcgactcct-3’
nicX-C76A-f	5’-gacggcctacgcaggcgacaccgcgctgaccggcaacctggc-3’
nicX-C76A-r	5’-gcggtgtcgcctgcgtaggccgtcatgtcgttgcccatggcgg-3’
nicX-C76Q-f	5’-gacggcctaccagggcgacaccgcgctgaccggcaacctggc-3’
nicX-C76Q-r	5’-gcggtgtcgccctggtaggccgtcatgtcgttgcccatggcgg-3’
nicX-C76E-f	5’-gacggcctacgaaggcgacaccgcgctgaccggcaacctggc-3’
nicX-C76E-r	5’-gcggtgtcgccttcgtaggccgtcatgtcgttgcccatggcgg-3’
nicX-L104A-f	5’-gatgctggcacactcgcccgagcaggagcagattctcaagac-3’
nicX-L104A-r	5’-ctcgggcgagtgtgccagcatcatggtgtcgacgaccaggtc-3’
nicX-H105A-f	5’-gatgctgctggcatcgcccgagcaggagcagattctcaagac-3’
nicX-H105A-r	5’-ctcgggcgatgccagcagcatcatggtgtcgacgaccaggtc-3’
nicX-H105F-f	5’-gatgctgctgttttcgcccgagcaggagcagattctcaagac-3’
nicX-H105F-r	5’-ctcgggcgaaaacagcagcatcatggtgtcgacgaccaggtc-3’
nicX-H105M-f	5’-gatgctgctgatgtcgcccgagcaggagcagattctcaagac-3’
nicX-H105M-r	5’-ctcgggcgacatcagcagcatcatggtgtcgacgaccaggtc-3’
nicX-V175F-f	5’-gtacccggcctttactgagtacggctatgccgacgaaccg-3’
nicX-V175F-r	5’-cgtactcagtaaaggccgggtactggccgagcggggcgtgg-3’
nicX-E177A-f	5’-gccgtgactgcatacggctatgccgacgaacc-3’
nicX-E177A-r	5’-ggttcgtcggcatagccgtatgcagtcacggc-3’
nicX-H189A-f	5’-ctgggacgcatggcccagcggctttctgttc-3’
nicX-H189A-r	5’-tgggccatgcgtcccagcgccccggttcgt-3’
nicX-E177A/H189-f	5’-ctgggacgcatggcccagcggctttctgttc-3’[a]
nicX-E177A/H189-r	5’-tgggccatgcgtcccagcgccccggttcgt-3’[a]
nicX-H265A-f	5’-cggcatctcggcaataggctggggcctgcagccgcgcgcgc-3’
nicX-H265A-r	5’-ccagcctattgccgagatgccgtatacctcggggtcgttgaag-3’
nicX-R293A-f	5’-gcatggatgccgcagcgttctacggc-3’
nicX-R293A-r	5’-gccgtagaacgctgcggcatccatgc-3’
nicX-S302A-f	5’-cttcctgttcgcaaccggccccaacaccgaggtcggcggc-3’
nicX-S302A-r	5’-ggggccggttgcgaacaggaagttgccgtagaacgcacg-3’
nicX-E308A-f	5’-gccccaacaccgcagtcggcggc-3’
nicX-E308A-r	5’-gccgccgactgcggtgttggggc-3’
nicX-H318A-f	5’-gaccccgtgcgcactggacatcccgctgcgcaactgcg-3’
nicX-H318A-r	5’-ggatgtccagtgcgcacggggtcttgcgcttgccgccg-3’
nicX-D320A-f	5’-gtgccacctggcaatcccgctgcgcaactgcgatatctacc-3’
nicX-D320A-r	5’-gcagcgggattgccaggtggcacggggtcttgcgcttgccg-3’

a. The NicX$^{E177A/H189}$ mutant was reconstructed using NicX^{E177A} as a template.

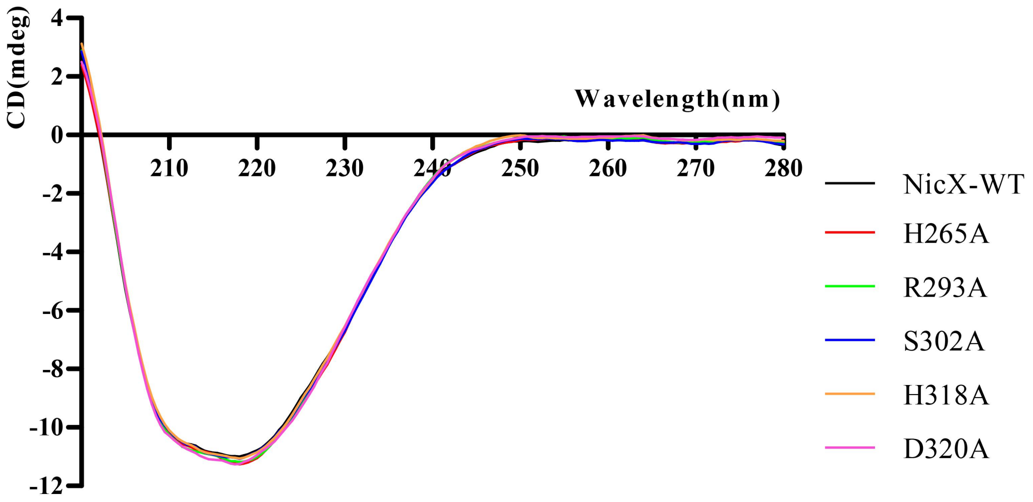

Circular dichrosim spectra of NicX and its mutants

Supplementary Figure 1. Circular dichroism spectra of NicX and its mutants. Variant proteins were 0.2 mg ml^{-1} in 20 mM NaH2PO4-Na2HPO4 buffer (pH 7.4), measured from 200 nm to 280 nm. (WT in black, H265A in red, R293A in green, S302A in blue, H318A in orange, D320A in pink).

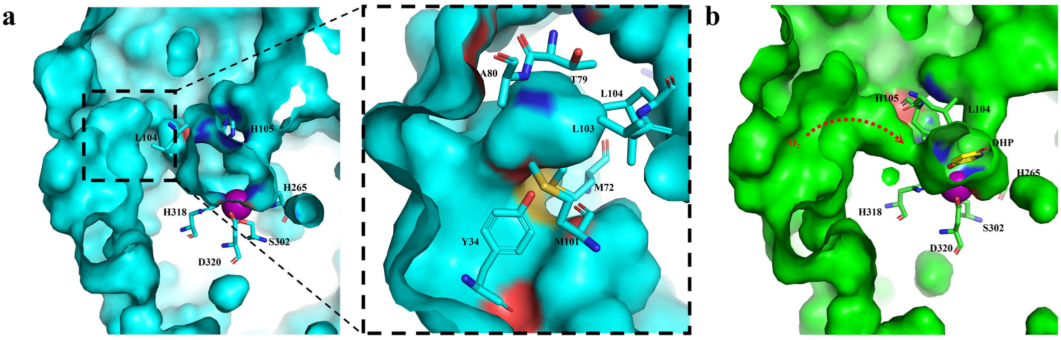

Supplementary Figure 2. A conformational change of Leu^{104}-His^{105} create a hydrophobic path that goes straight to the active center of ferrous ion. (**a**) In resting subunits or NFM bound subunits, the hydrophobic path is blocked by residue Leu^{104}, the dotted box zoomed in shows pockets made of hydrophobic amino acids. (**b**) In DHP bound subunits, conformational change of Leu^{104}-His^{105} result in hydrophobic pockets connect with the active center of ferrous ion, and thus may act as a channel for O_2.

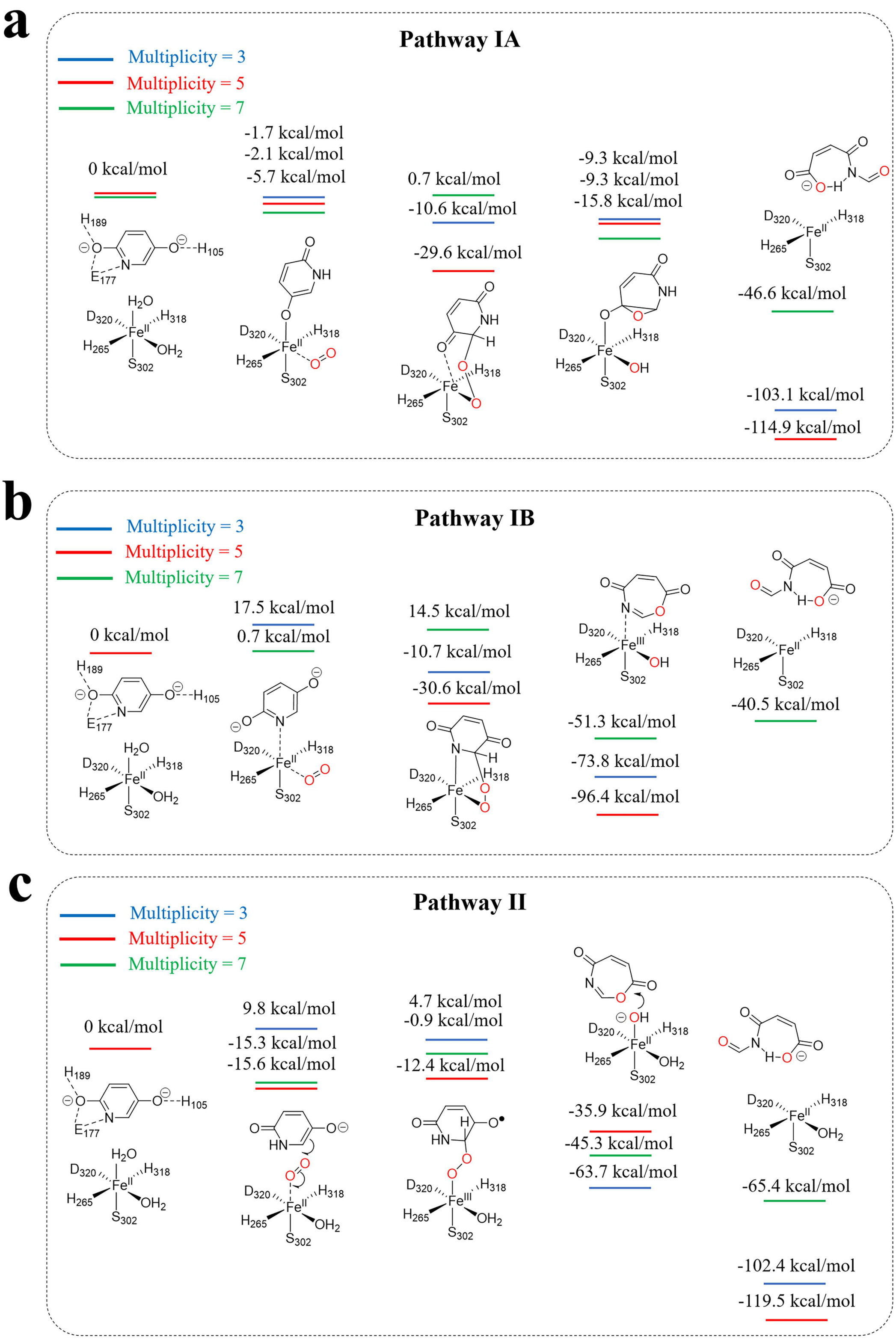

Supplementary Figure 3. The calculated energetics of pathways IA, IB, and II, where the different multiplicities were considered and the lowest energies were plotted. (**a**)

The optimized energies of pathway IA, the oxygen is coordinated on the axis opposite to Asp^{320} and the O5 of DHP is coordinated to Fe. (**b**) The optimized energies of pathway IB, the oxygen is coordinated on the axis opposite to Asp^{320} and the N atom of DHP is coordinated to Fe. (**c**) The optimized energies of pathway II, the oxygen is coordinated on the axis opposite to Ser^{302} and DHP is attacked by oxygen. Different spin multiplicity is labeled with different colors (Blue: Triplet, Red: Quintet, Green: Septet). The reactant complex is considered as zero energy point in every pathway, and the energies of the following structures are obtained by subtracting the reactants.

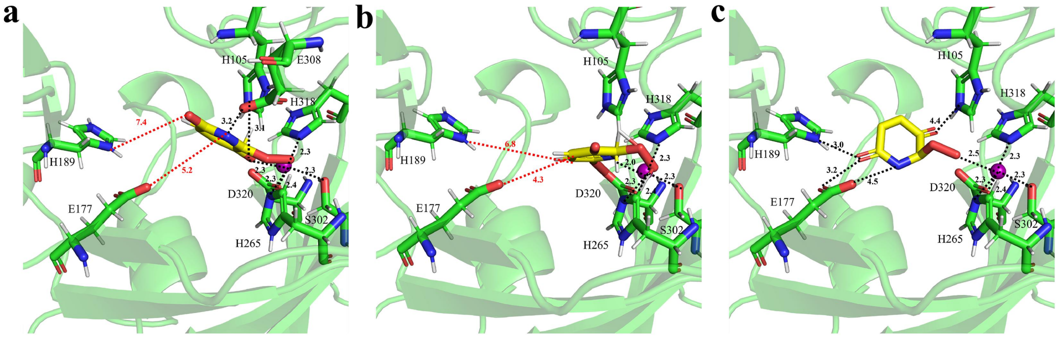

Supplementary Figure 4. The calculated structures of critical peroxide intermediates in pathways IA (**a**), IB (**b**), and II(**c**), constructed with the crystal structures with substrate DHP and product NMF.

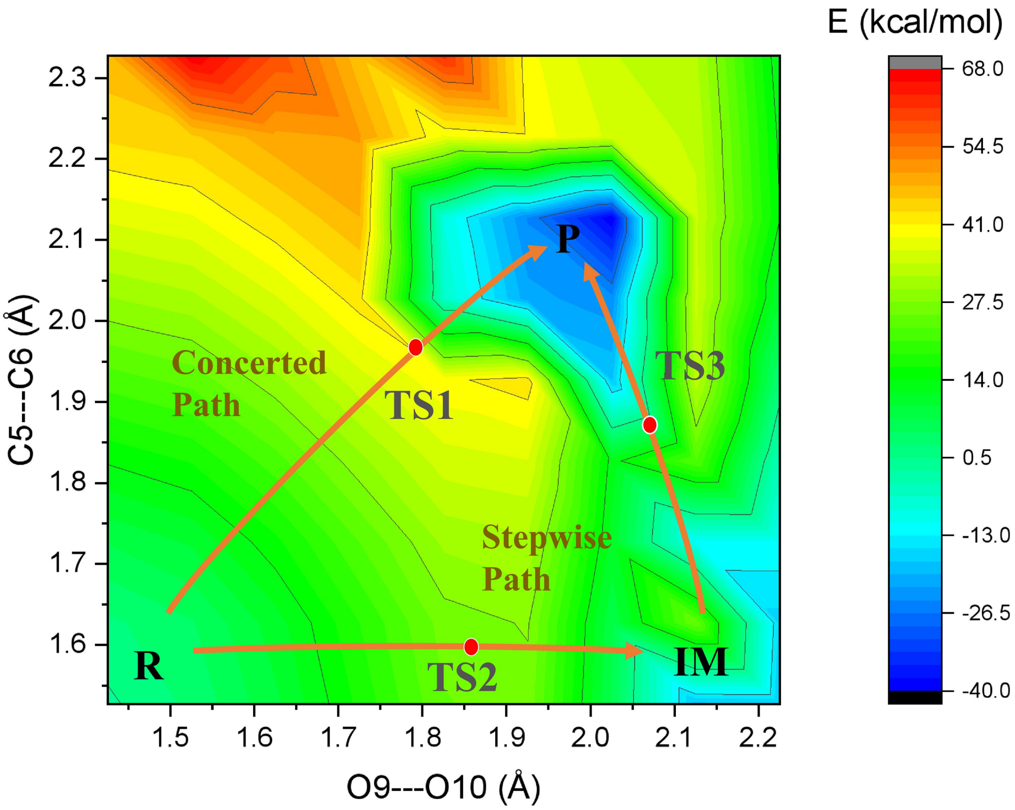

Supplementary Figure 5. Potential energy surface of O9-O10 and C5-C6 bonds in the transition state. R: peroxide intermediate, P: 7-membered-ring lactone; TS1: concerted transition state, TS2: O-O cleavage transition state, TS3: C-C cleavage transition state, IM: O-O cleavage product.
