# Supplementary material for: Structure-guided insights into heterocyclic ring-cleavage catalysis of the non-heme Fe (II) dioxygenase NicX
Source: Nat Commun. 2021 Feb 26;12:1301. doi: 10.1038/s41467-021-21567-9 (PMC7910607; doi:10.1038/s41467-021-21567-9)
Supplement: Supplementary file 3 — Description of Additional Supplementary Files [file 41467_2021_21567_MOESM3_ESM.doc]

Supplementary Movie 1

Nimation of the Leu^104^ and His^105^ motion from the state of substrate-binding to state of product-binding.
