# Supplementary material for: Structure-guided insights into heterocyclic ring-cleavage catalysis of the non-heme Fe (II) dioxygenase NicX
Source: Nat Commun. 2021 Feb 26;12:1301. doi: 10.1038/s41467-021-21567-9 (PMC7910607; doi:10.1038/s41467-021-21567-9)
Supplement: Supplementary file 6 — Source Data [file 41467_2021_21567_MOESM6_ESM.zip › Source data/Source data 1-D_1300017919_val-report-full_P1-7CN3.pdf]

Preliminary Full wwPDB X-ray Structure Validation Report (i)

Jul 30, 2020 – 10:36 AM JST

Deposition ID : D_1300017919

This is a Preliminary Full wwPDB X-ray Structure Validation Report.

This report is produced by the wwPDB Deposition System during initial deposition but before annotation of the structure.

We welcome your comments at *validation@mail.wwpdb.org*
A user guide is available at
https://www.wwpdb.org/validation/2017/XrayValidationReportHelp
with specific help available everywhere you see the (i) symbol.

PRELIMINARY VALIDATION REPORT

The following versions of software and data (see references (i)) were used in the production of this report:

MolProbity	:	4.02b-467
Mogul	:	1.8.5 (274361), CSD as541be (2020)
Xtriage (Phenix)	:	1.13
EDS	:	2.13.1
buster-report	:	1.1.7 (2018)
Percentile statistics	:	20191225.v01 (using entries in the PDB archive December 25th 2019)
Refmac	:	5.8.0158
CCP4	:	7.0.044 (Gargrove)
Ideal geometry (proteins)	:	Engh & Huber (2001)
Ideal geometry (DNA, RNA)	:	Parkinson et al. (1996)
Validation Pipeline (wwPDB-VP)	:	2.13.1

1 Overall quality at a glance (i)

The following experimental techniques were used to determine the structure:
X-RAY DIFFRACTION

The reported resolution of this entry is 2.20 Å.

Percentile scores (ranging between 0-100) for global validation metrics of the entry are shown in the following graphic. The table shows the number of entries on which the scores are based.

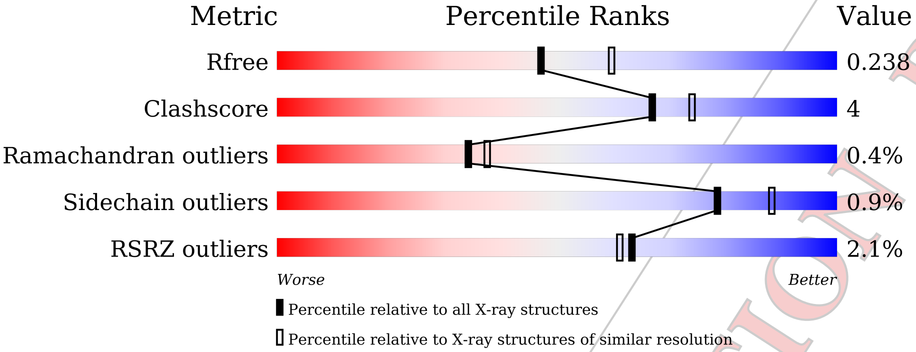

Metric	Whole archive (#Entries)	Similar resolution (#Entries, resolution range(Å))
R_{free}	130704	4898 (2.20-2.20)
Clashscore	141614	5594 (2.20-2.20)
Ramachandran outliers	138981	5503 (2.20-2.20)
Sidechain outliers	138945	5504 (2.20-2.20)
RSRZ outliers	127900	4800 (2.20-2.20)

The table below summarises the geometric issues observed across the polymeric chains and their fit to the electron density. The red, orange, yellow and green segments on the lower bar indicate the fraction of residues that contain outliers for >=3, 2, 1 and 0 types of geometric quality criteria respectively. A grey segment represents the fraction of residues that are not modelled. The numeric value for each fraction is indicated below the corresponding segment, with a dot representing fractions <=5% The upper red bar (where present) indicates the fraction of residues that have poor fit to the electron density. The numeric value is given above the bar.

Mol	Chain	Length	Quality of chain
1	A	348	% / 92% 8% •
1	B	348	2% / 94% 6%
1	E	348	3% / 90% 9% •
1	F	348	3% / 91% 8%
2	C	349	% / 92% 8%
2	D	349	3% / 91% 9% •

PRELIMINARY VALIDATION REPORT

The following table lists non-polymeric compounds, carbohydrate monomers and non-standard residues in protein, DNA, RNA chains that are outliers for geometric or electron-density-fit criteria:

Mol	Type	Chain	Res	Chirality	Geometry	Clashes	Electron density
6	EDO	S	5	-	-	X	-

PRELIMINARY VALIDATION REPORT

2 Entry composition (i)

There are 8 unique types of molecules in this entry. The entry contains 17618 atoms, of which 0 are hydrogens and 0 are deuteriums.

In the tables below, the ZeroOcc column contains the number of atoms modelled with zero occupancy, the AltConf column contains the number of residues with at least one atom in alternate conformation and the Trace column contains the number of residues modelled with at most 2 atoms.

- Molecule 1 is a protein.

Mol	Chain	Residues	Atoms					ZeroOcc	AltConf	Trace
1	A	348	Total 2750	C 1734	N 480	O 520	S 16	0	3	0
1	B	348	Total 2739	C 1728	N 476	O 519	S 16	0	2	0
1	E	348	Total 2760	C 1741	N 481	O 522	S 16	0	4	0
1	F	348	Total 2763	C 1743	N 483	O 521	S 16	0	4	0

- Molecule 2 is a protein.

Mol	Chain	Residues	Atoms					ZeroOcc	AltConf	Trace
2	C	349	Total 2770	C 1746	N 486	O 522	S 16	0	4	0
2	D	349	Total 2788	C 1756	N 489	O 527	S 16	0	6	0

- Molecule 3 is FE (II) ION (three-letter code: FE2) (formula: Fe).

Mol	Chain	Residues	Atoms		ZeroOcc	AltConf
3	A	1	Total 1	Fe 1	0	0
3	B	1	Total 1	Fe 1	0	0
3	C	1	Total 1	Fe 1	0	0
3	D	1	Total 1	Fe 1	0	0
3	E	1	Total 1	Fe 1	0	0
3	F	1	Total 1	Fe 1	0	0

PRELIMINARY VALIDATION REPORT

- Molecule 4 is a ligand with the chemical component id NFM but its atom names do not match the existing wwPDB Chemical Component Dictionary definition for NFM. Consequently no firm identification of ligand chemistry can be made. Once the structure is annotated then an identification and diagram will be given here.

Mol	Chain	Residues	Atoms	ZeroOcc	AltConf
4	A	1	Total C N O<br>10 5 1 4	0	0
4	B	1	Total C N O<br>10 5 1 4	0	0

- Molecule 5 is a ligand with the chemical component id DHP but its atom names do not match the existing wwPDB Chemical Component Dictionary definition for DHP. Consequently no firm identification of ligand chemistry can be made. Once the structure is annotated then an identification and diagram will be given here.

Mol	Chain	Residues	Atoms	ZeroOcc	AltConf
5	C	1	Total C N O<br>8 5 1 2	0	0
5	D	1	Total C N O<br>8 5 1 2	0	0
5	E	1	Total C N O<br>8 5 1 2	0	0
5	F	1	Total C N O<br>8 5 1 2	0	0

- Molecule 6 is 1,2-ETHANEDIOL (three-letter code: EDO) (formula: $C_2H_6O_2$).

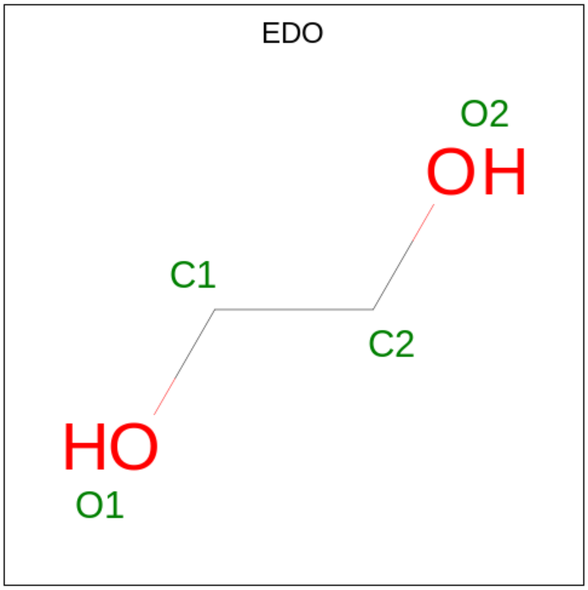

PRELIMINARY VALIDATION REPORT

Mol	Chain	Residues	Atoms	ZeroOcc	AltConf
6	S	1	Total C O<br>4 2 2	0	0
6	S	1	Total C O<br>4 2 2	0	0
6	S	1	Total C O<br>4 2 2	0	0
6	S	1	Total C O<br>4 2 2	0	0
6	S	1	Total C O<br>4 2 2	0	0
6	S	1	Total C O<br>4 2 2	0	0
6	S	1	Total C O<br>4 2 2	0	0
6	S	1	Total C O<br>4 2 2	0	0

- Molecule 7 is TRIETHYLENE GLYCOL (three-letter code: PGE) (formula: $C_6H_{14}O_4$).

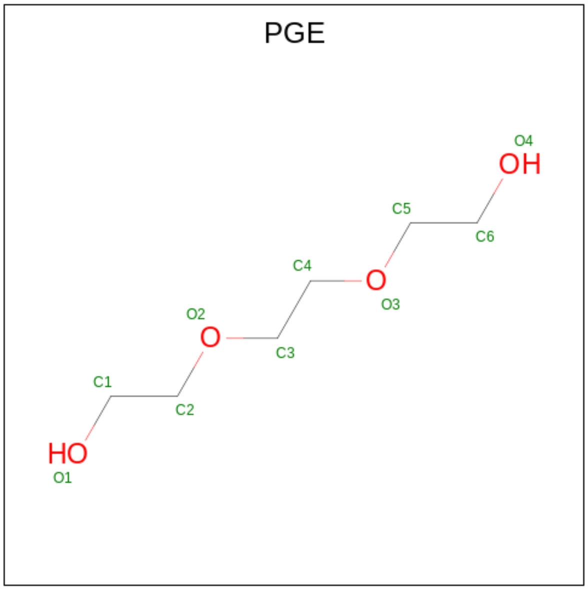

Mol	Chain	Residues	Atoms	ZeroOcc	AltConf
7	S	1	Total C O<br>10 6 4	0	0
7	S	1	Total C O<br>10 6 4	0	0

- Molecule 8 is water.

PRELIMINARY VALIDATION REPORT

Mol	Chain	Residues	Atoms		ZeroOcc	AltConf
			Total	O		
8	W	938	938	938	0	0

PRELIMINARY VALIDATION REPORT

3 Residue-property plots (i)

These plots are drawn for all protein, RNA, DNA and oligosaccharide chains in the entry. The first graphic for a chain summarises the proportions of the various outlier classes displayed in the second graphic. The second graphic shows the sequence view annotated by issues in geometry and electron density. Residues are color-coded according to the number of geometric quality criteria for which they contain at least one outlier: green = 0, yellow = 1, orange = 2 and red = 3 or more. A red dot above a residue indicates a poor fit to the electron density (RSRZ > 2). Stretches of 2 or more consecutive residues without any outlier are shown as a green connector. Residues present in the sample, but not in the model, are shown in grey.

• Molecule 1:

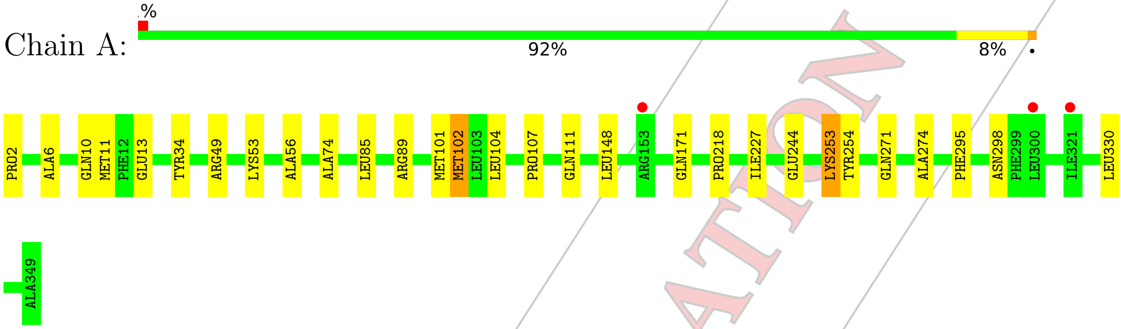

• Molecule 1:

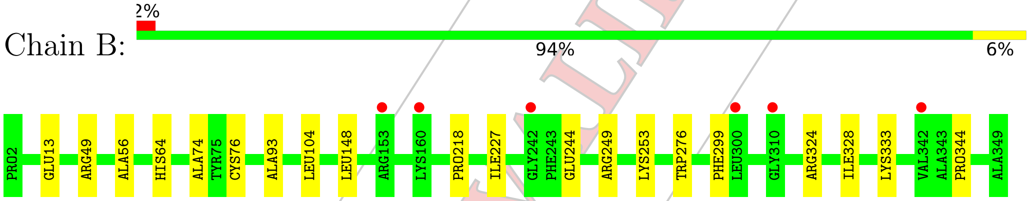

• Molecule 1:

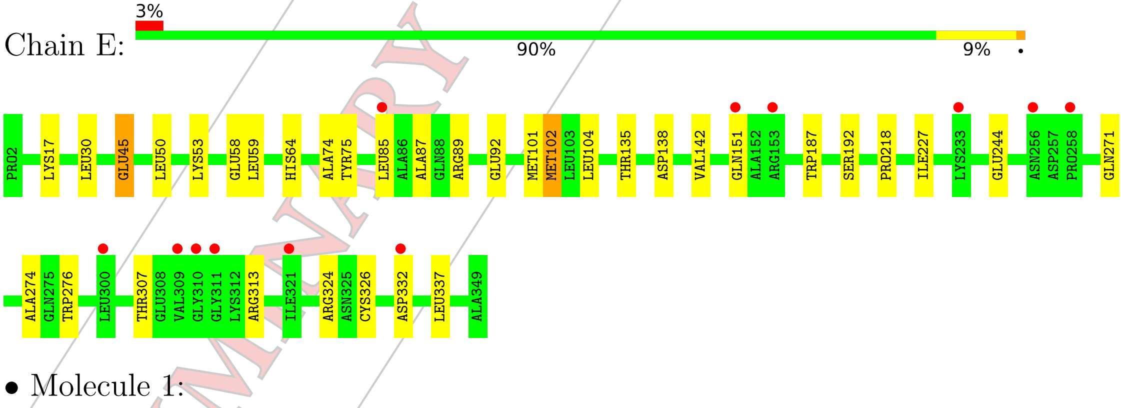

• Molecule 1:

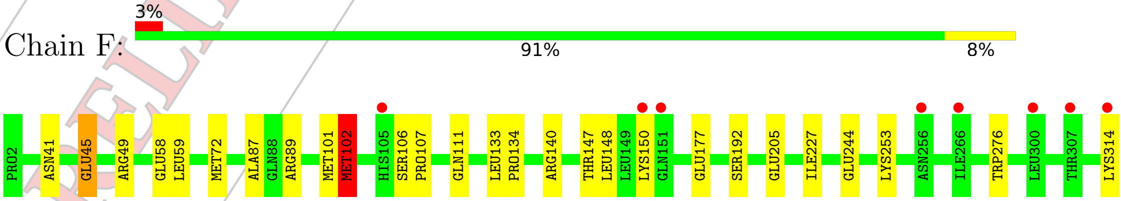

PRELIMINARY VALIDATION REPORT

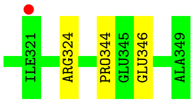

• Molecule 2:

Chain C:

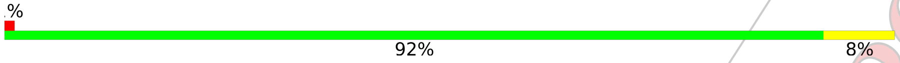

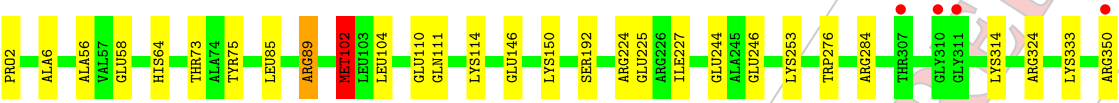

• Molecule 2:

Chain D:

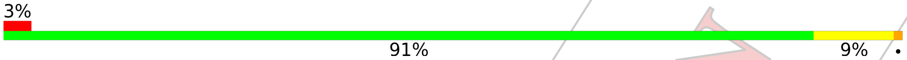

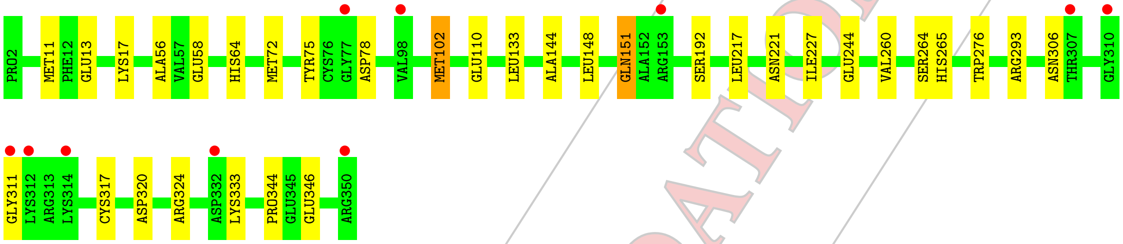

PRELIMINARY VALIDATION REPORT

4 Data and refinement statistics ⓘ

Property	Value	Source
Space group	P 21 21 2	Depositor
Cell constants<br>a, b, c, α, β, γ	126.67Å 145.51Å 118.98Å<br>90.00° 90.00° 90.00°	Depositor
Resolution (Å)	27.59 – 2.20<br>27.59 – 2.18	Depositor<br>EDS
% Data completeness<br>(in resolution range)	99.6 (27.59-2.20)<br>98.7 (27.59-2.18)	Depositor<br>EDS
R_{merge}	(Not available)	Depositor
R_{sym}	(Not available)	Depositor
$< I/\sigma(I) >$ [1]	1.90 (at 2.18Å)	Xtriage
Refinement program	PHENIX 1.14_3260	Depositor
R, R_{free}	0.183 , 0.238<br>0.184 , 0.238	Depositor<br>DCC
R_{free} test set	5645 reflections (5.00%)	wwPDB-VP
Wilson B-factor (Å^2)	30.9	Xtriage
Anisotropy	0.053	Xtriage
Bulk solvent k_{sol}(e/Å^3), B_{sol}(Å^2)	0.32 , 36.7	EDS
L-test for twinning[2]	$< \|L\| > = 0.50$, $< L^2 > = 0.34$	Xtriage
Estimated twinning fraction	No twinning to report.	Xtriage
F_o,F_c correlation	0.97	EDS
Total number of atoms	17618	wwPDB-VP
Average B, all atoms (Å^2)	35.0	wwPDB-VP

Xtriage's analysis on translational NCS is as follows: *The analyses of the Patterson function reveals a significant off-origin peak that is 50.65 % of the origin peak, indicating pseudo-translational symmetry. The chance of finding a peak of this or larger height randomly in a structure without pseudo-translational symmetry is equal to 6.2726e-05. The detected translational NCS is most likely also responsible for the elevated intensity ratio.*

[1] Intensities estimated from amplitudes.
[2] Theoretical values of $< |L| >$, $< L^2 >$ for acentric reflections are 0.5, 0.333 respectively for untwinned datasets, and 0.375, 0.2 for perfectly twinned datasets.

PRELIMINARY VALIDATION REPORT

PRELIMINARY VALIDATION REPORT

5 Model quality (i)

5.1 Standard geometry (i)

Bond lengths and bond angles in the following residue types are not validated in this section: NFM, PGE, FE2, EDO, DHP

The Z score for a bond length (or angle) is the number of standard deviations the observed value is removed from the expected value. A bond length (or angle) with $|Z| > 5$ is considered an outlier worth inspection. RMSZ is the root-mean-square of all Z scores of the bond lengths (or angles).

Mol	Chain	Bond lengths		Bond angles	
		RMSZ	$\#\|Z\| >5$	RMSZ	$\#\|Z\| >5$
1	A	0.40	0/2815	0.56	0/3824
1	B	0.41	0/2804	0.57	0/3810
1	E	0.41	0/2827	0.57	0/3841
1	F	0.41	0/2830	0.58	0/3844
2	C	0.44	0/2837	0.58	0/3854
2	D	0.41	0/2855	0.58	0/3878
All	All	0.42	0/16968	0.57	0/23051

There are no bond length outliers.

There are no bond angle outliers.

There are no chirality outliers.

There are no planarity outliers.

5.2 Too-close contacts (i)

In the following table, the Non-H and H(model) columns list the number of non-hydrogen atoms and hydrogen atoms in the chain respectively. The H(added) column lists the number of hydrogen atoms added and optimized by MolProbity. The Clashes column lists the number of clashes within the asymmetric unit, whereas Symm-Clashes lists symmetry related clashes.

Mol	Chain	Non-H	H(model)	H(added)	Clashes	Symm-Clashes
1	A	2750	0	2675	22	0
1	B	2739	0	2663	15	0
1	E	2760	0	2675	26	0
1	F	2763	0	2683	23	1
2	C	2770	0	2688	20	1
2	D	2788	0	2700	30	0
3	A	1	0	0	0	0

Continued on next page...

Continued from previous page...

Mol	Chain	Non-H	H(model)	H(added)	Clashes	Symm-Clashes
3	B	1	0	0	0	0
3	C	1	0	0	0	0
3	D	1	0	0	0	0
3	E	1	0	0	0	0
3	F	1	0	0	0	0
4	A	10	0	0	0	0
4	B	10	0	0	0	0
5	C	8	0	0	1	0
5	D	8	0	0	1	0
5	E	8	0	0	1	0
5	F	8	0	0	1	0
6	S	32	0	48	5	0
7	S	20	0	28	1	0
8	W	938	0	0	27	0
All	All	17618	0	16160	128	1

The all-atom clashscore is defined as the number of clashes found per 1000 atoms (including hydrogen atoms). The all-atom clashscore for this structure is 4.

All (128) close contacts within the same asymmetric unit are listed below, sorted by their clash magnitude.

Atom-1	Atom-2	Interatomic distance (Å)	Clash overlap (Å)
5:C:2001:DHP:OB	8:W:939:HOH:O	1.83	0.95
2:D:72:MET:HB2	2:D:102:MET:HE3	1.49	0.93
5:D:2001:DHP:OB	8:W:940:HOH:O	1.89	0.89
2:D:333:LYS:NZ	8:W:855:HOH:O	2.10	0.83
2:D:72:MET:CB	2:D:102:MET:HE3	2.10	0.80
2:D:17:LYS:NZ	8:W:274:HOH:O	2.14	0.78
1:A:253:LYS:NZ	8:W:745:HOH:O	2.20	0.74
5:E:2001:DHP:OB	8:W:938:HOH:O	2.07	0.71
2:C:227:ILE:HD11	2:C:244:GLU:HB3	1.72	0.71
2:D:72:MET:C	2:D:102:MET:HE1	2.10	0.71
2:D:227:ILE:HD11	2:D:244:GLU:HB3	1.72	0.70
1:B:227:ILE:HD11	1:B:244:GLU:HB3	1.74	0.70
2:D:221[B]:ASN:ND2	8:W:731:HOH:O	2.23	0.68
1:E:151:GLN:NE2	8:W:524:HOH:O	2.26	0.67
1:E:64:HIS:HB3	6:S:5:EDO:H21	1.76	0.67
2:D:320:ASP:OD1	8:W:940:HOH:O	2.14	0.65
1:A:227:ILE:HD11	1:A:244:GLU:HB3	1.77	0.65
1:A:10:GLN:NE2	8:W:427:HOH:O	2.30	0.65
2:C:225:GLU:HG2	2:C:244:GLU:OE1	1.98	0.65

Continued on next page...

PRELIMINARY VALIDATION REPORT

Continued from previous page...

Atom-1	Atom-2	Interatomic distance (Å)	Clash overlap (Å)
2:D:72:MET:HB2	2:D:102:MET:CE	2.26	0.64
1:F:45[B]:GLU:OE2	8:W:914:HOH:O	2.15	0.63
1:E:227:ILE:HD11	1:E:244:GLU:HB3	1.81	0.62
2:D:293:ARG:NE	2:D:320:ASP:OD2	2.30	0.62
1:B:148:LEU:HD22	1:B:344:PRO:HG3	1.82	0.60
1:E:92:GLU:O	8:W:746:HOH:O	2.16	0.60
1:A:11:MET:HG3	6:S:7:EDO:H22	1.83	0.60
1:F:227:ILE:HD11	1:F:244:GLU:HB3	1.83	0.59
1:A:148:LEU:HG	1:A:330:LEU:HD12	1.85	0.59
1:E:17:LYS:HE2	1:E:50:LEU:HD22	1.85	0.58
1:F:45[B]:GLU:OE1	8:W:772:HOH:O	2.17	0.57
1:E:75:TYR:CZ	6:S:5:EDO:H22	2.40	0.57
1:F:276:TRP:CD1	1:F:324:ARG:HD2	2.39	0.57
1:A:107:PRO:O	1:A:111:GLN:HG3	2.06	0.56
1:B:93:ALA:HA	1:F:89:ARG:NH2	2.21	0.56
1:B:276:TRP:NE1	1:B:324:ARG:HD2	2.21	0.55
1:B:56:ALA:HB3	1:F:58:GLU:HB3	1.89	0.55
2:C:85:LEU:HD12	2:C:89:ARG:HH12	1.72	0.55
1:A:2:PRO:HD2	8:W:901:HOH:O	2.07	0.54
2:C:85:LEU:HD12	2:C:89:ARG:NH1	2.22	0.54
1:E:75:TYR:OH	6:S:5:EDO:H22	2.08	0.54
2:C:6:ALA:HB2	1:E:45[A]:GLU:HG3	1.90	0.53
1:F:177:GLU:OE1	5:F:2001:DHP:OH	2.25	0.53
1:B:249:ARG:HA	8:W:724:HOH:O	2.09	0.53
1:F:41:ASN:O	1:F:45[A]:GLU:HG2	2.09	0.53
2:D:148:LEU:O	2:D:151[A]:GLN:HG3	2.08	0.53
1:A:171:GLN:OE1	8:W:754:HOH:O	2.18	0.53
2:D:13:GLU:OE2	1:F:49:ARG:NH1	2.36	0.52
2:D:13:GLU:O	2:D:17:LYS:HE3	2.09	0.52
2:D:110:GLU:HG2	8:W:897:HOH:O	2.10	0.52
1:B:276:TRP:CD1	1:B:324:ARG:HD2	2.44	0.52
1:E:307:THR:HG23	1:E:313:ARG:O	2.10	0.51
2:D:276:TRP:CD1	2:D:324:ARG:HD2	2.45	0.51
1:E:326:CYS:HB2	8:W:69:HOH:O	2.09	0.51
2:D:11:MET:HG2	2:D:133:LEU:HD13	1.92	0.50
2:C:276:TRP:CD1	2:C:324:ARG:HD2	2.46	0.50
2:D:260:VAL:CG1	2:D:317:CYS:HB3	2.42	0.50
2:D:144:ALA:HB1	2:D:346[A]:GLU:HG2	1.95	0.49
2:C:110:GLU:OE1	8:W:892:HOH:O	2.19	0.49
2:D:72:MET:CB	2:D:102:MET:CE	2.85	0.49
1:F:101:MET:O	1:F:102:MET:HB3	2.12	0.48

Continued on next page...

PRELIMINARY VALIDATION REPORT

Continued from previous page...

Atom-1	Atom-2	Interatomic distance (Å)	Clash overlap (Å)
1:F:276:TRP:NE1	1:F:324:ARG:HD2	2.28	0.48
1:B:74:ALA:HB2	1:B:218:PRO:HG3	1.95	0.48
1:A:49:ARG:NH2	1:B:13:GLU:OE1	2.47	0.48
1:E:271:GLN:HG3	1:E:274:ALA:H	1.78	0.48
1:F:140[B]:ARG:NH1	1:F:346:GLU:HG2	2.29	0.48
1:F:148:LEU:HD12	1:F:344:PRO:HG3	1.94	0.47
1:F:253:LYS:HE2	8:W:559:HOH:O	2.13	0.47
1:E:276:TRP:NE1	1:E:324:ARG:HD2	2.30	0.47
1:A:85:LEU:HD11	1:A:89[A]:ARG:CZ	2.45	0.47
2:C:111:GLN:O	2:C:114:LYS:HB2	2.15	0.47
1:E:17:LYS:HD3	1:E:17:LYS:HA	1.79	0.47
2:D:72:MET:HB3	2:D:102:MET:HE3	1.95	0.47
1:F:147:THR:HA	1:F:150:LYS:CD	2.45	0.47
2:D:260:VAL:HG12	2:D:317:CYS:HB3	1.96	0.47
1:A:101:MET:O	1:A:102:MET:HB3	2.14	0.47
1:B:299:PHE:HB2	1:B:328:ILE:HD11	1.96	0.47
2:C:56:ALA:HB3	2:D:58:GLU:HB3	1.96	0.46
1:E:53:LYS:HD3	1:E:53:LYS:N	2.29	0.46
1:A:271:GLN:HG3	1:A:274:ALA:H	1.80	0.46
2:D:148:LEU:HD22	2:D:344:PRO:HG3	1.97	0.46
1:A:253:LYS:HD2	1:A:254:TYR:N	2.30	0.46
2:C:333:LYS:O	2:C:333:LYS:HG3	2.15	0.46
2:D:276:TRP:NE1	2:D:324:ARG:HD2	2.31	0.46
1:F:140[B]:ARG:HH12	1:F:346:GLU:HG2	1.79	0.46
2:C:246:GLU:OE2	1:F:324:ARG:NH2	2.34	0.45
2:D:264:SER:OG	2:D:265:HIS:N	2.48	0.45
1:A:74:ALA:HB2	1:A:218:PRO:HG3	1.98	0.45
2:C:146:GLU:HG2	2:C:150:LYS:HE2	1.97	0.45
1:F:147:THR:HA	1:F:150:LYS:HD2	1.97	0.45
1:A:53:LYS:NZ	8:W:120:HOH:O	2.17	0.45
1:A:85:LEU:O	1:A:89[B]:ARG:HG3	2.16	0.45
1:B:104:LEU:HD23	1:B:104:LEU:HA	1.79	0.45
1:E:64:HIS:CB	6:S:5:EDO:H21	2.46	0.45
1:F:148:LEU:HD23	1:F:148:LEU:HA	1.75	0.44
2:D:64:HIS:CE1	2:D:78:ASP:HB2	2.53	0.44
1:B:253:LYS:HD2	1:B:253:LYS:HA	1.80	0.44
2:C:114:LYS:HA	2:C:114:LYS:HD3	1.78	0.44
1:A:104:LEU:H	1:A:104:LEU:HD22	1.82	0.44
1:B:76:CYS:HA	1:B:104:LEU:HD13	1.99	0.43
1:F:59:LEU:HD21	1:F:87:ALA:HB1	2.00	0.43
1:A:6:ALA:O	1:A:10:GLN:NE2	2.52	0.43

Continued on next page...

PRELIMINARY VALIDATION REPORT

Continued from previous page...

Atom-1	Atom-2	Interatomic distance (Å)	Clash overlap (Å)
1:A:13:GLU:OE2	1:B:49:ARG:NH2	2.48	0.43
1:E:85:LEU:HB2	8:W:595:HOH:O	2.17	0.43
1:A:34:TYR:CE2	2:C:2:PRO:HG2	2.54	0.43
1:A:56:ALA:HB3	1:E:58:GLU:HB3	2.00	0.43
1:B:64:HIS:HD2	8:W:803:HOH:O	2.02	0.43
2:C:253:LYS:HA	7:S:9:PGE:H6	2.01	0.43
2:C:104:LEU:HD12	8:W:777:HOH:O	2.17	0.43
1:E:104:LEU:HD13	1:E:187:TRP:CE2	2.53	0.43
1:E:276:TRP:CD1	1:E:324:ARG:HD2	2.54	0.43
2:D:217:LEU:HB2	2:D:320:ASP:HB2	2.01	0.42
1:E:101:MET:O	1:E:102:MET:HB3	2.18	0.42
1:E:135:THR:HG22	8:W:550:HOH:O	2.19	0.42
1:A:295:PHE:CE1	1:A:298:ASN:HB3	2.55	0.42
1:E:30:LEU:HD11	1:E:101:MET:HE3	2.01	0.42
2:C:224:ARG:HD3	8:W:703:HOH:O	2.19	0.42
1:E:59:LEU:HD21	1:E:87:ALA:HB1	2.02	0.42
1:E:337:LEU:HA	1:E:337:LEU:HD23	1.90	0.41
1:F:107:PRO:O	1:F:111:GLN:HG3	2.20	0.41
1:F:72:MET:HE1	8:W:901:HOH:O	2.20	0.41
1:E:74:ALA:HB2	1:E:218:PRO:HG3	2.01	0.41
2:C:58:GLU:HB3	2:D:56:ALA:HB3	2.00	0.41
2:C:73:THR:HG22	2:C:102:MET:CE	2.50	0.41
1:E:138:ASP:O	1:E:142:VAL:HG23	2.21	0.41
2:D:64:HIS:CG	2:D:75:TYR:CD1	3.09	0.41
2:C:64:HIS:CG	2:C:75:TYR:CD1	3.09	0.41
1:F:133:LEU:HA	1:F:134:PRO:HD3	1.88	0.41
2:D:306:ASN:OD1	2:D:311:GLY:HA3	2.21	0.40

All (1) symmetry-related close contacts are listed below. The label for Atom-2 includes the symmetry operator and encoded unit-cell translations to be applied.

Atom-1	Atom-2	Interatomic distance (Å)	Clash overlap (Å)
2:C:350:ARG:NH2	1:F:205:GLU:OE1[4_457]	2.13	0.07

5.3 Torsion angles (i)

5.3.1 Protein backbone (i)

In the following table, the Percentiles column shows the percent Ramachandran outliers of the chain as a percentile score with respect to all X-ray entries followed by that with respect to entries

PRELIMINARY VALIDATION REPORT

of similar resolution.

The Analysed column shows the number of residues for which the backbone conformation was analysed, and the total number of residues.

Mol	Chain	Analysed	Favoured	Allowed	Outliers	Percentiles	
1	A	349/348 (100%)	334 (96%)	14 (4%)	1 (0%)	41	46
1	B	348/348 (100%)	329 (94%)	19 (6%)	0	100	100
1	E	350/348 (101%)	335 (96%)	14 (4%)	1 (0%)	41	46
1	F	350/348 (101%)	334 (95%)	14 (4%)	2 (1%)	25	26
2	C	351/349 (101%)	339 (97%)	10 (3%)	2 (1%)	25	26
2	D	353/349 (101%)	335 (95%)	16 (4%)	2 (1%)	25	26
All	All	2101/2090 (100%)	2006 (96%)	87 (4%)	8 (0%)	34	37

All (8) Ramachandran outliers are listed below:

Mol	Chain	Res	Type
1	A	102	MET
2	C	102	MET
2	D	192	SER
1	E	192	SER
2	C	192	SER
2	D	102	MET
1	F	102	MET
1	F	192	SER

5.3.2 Protein sidechains ⓘ

In the following table, the Percentiles column shows the percent sidechain outliers of the chain as a percentile score with respect to all X-ray entries followed by that with respect to entries of similar resolution.

The Analysed column shows the number of residues for which the sidechain conformation was analysed, and the total number of residues.

Mol	Chain	Analysed	Rotameric	Outliers	Percentiles	
1	A	290/287 (101%)	289 (100%)	1 (0%)	92	97
1	B	289/287 (101%)	288 (100%)	1 (0%)	92	97
1	E	291/287 (101%)	286 (98%)	5 (2%)	60	74
1	F	291/287 (101%)	286 (98%)	5 (2%)	60	74
2	C	292/288 (101%)	288 (99%)	4 (1%)	67	80

Continued on next page...

PRELIMINARY VALIDATION REPORT

Continued from previous page...

Mol	Chain	Analysed	Rotameric	Outliers	Percentiles	
2	D	294/288 (102%)	292 (99%)	2 (1%)	84	91
All	All	1747/1724 (101%)	1729 (99%)	18 (1%)	78	86

All (18) residues with a non-rotameric sidechain are listed below:

Mol	Chain	Res	Type
1	A	253	LYS
1	B	333	LYS
2	C	89	ARG
2	C	102	MET
2	C	284	ARG
2	C	314	LYS
2	D	151[A]	GLN
2	D	151[B]	GLN
1	E	45[A]	GLU
1	E	45[B]	GLU
1	E	89	ARG
1	E	102	MET
1	E	332	ASP
1	F	45[A]	GLU
1	F	45[B]	GLU
1	F	102	MET
1	F	106	SER
1	F	314	LYS

Some sidechains can be flipped to improve hydrogen bonding and reduce clashes. All (2) such sidechains are listed below:

Mol	Chain	Res	Type
1	A	10	GLN
2	D	64	HIS

5.3.3 RNA (i)

There are no RNA molecules in this entry.

5.4 Non-standard residues in protein, DNA, RNA chains (i)

There are no non-standard protein/DNA/RNA residues in this entry.

PRELIMINARY VALIDATION REPORT

PRELIMINARY VALIDATION REPORT

5.5 Carbohydrates

There are no monosaccharides in this entry.

5.6 Ligand geometry

Of 22 ligands modelled in this entry, 6 could not be matched to an existing wwPDB Chemical Component Dictionary definition at this stage and 6 are monoatomic - leaving 10 for Mogul analysis.

In the following table, the Counts columns list the number of bonds (or angles) for which Mogul statistics could be retrieved, the number of bonds (or angles) that are observed in the model and the number of bonds (or angles) that are defined in the Chemical Component Dictionary. The Link column lists molecule types, if any, to which the group is linked. The Z score for a bond length (or angle) is the number of standard deviations the observed value is removed from the expected value. A bond length (or angle) with $|Z| > 2$ is considered an outlier worth inspection. RMSZ is the root-mean-square of all Z scores of the bond lengths (or angles).

Mol	Type	Chain	Res	Link	Bond lengths			Bond angles		
					Counts	RMSZ	$\#\|Z\| > 2$	Counts	RMSZ	$\#\|Z\| > 2$
6	EDO	S	2	-	3,3,3	0.51	0	2,2,2	0.28	0
6	EDO	S	7	-	3,3,3	0.52	0	2,2,2	0.20	0
7	PGE	S	9	-	9,9,9	0.42	0	8,8,8	0.39	0
6	EDO	S	3	-	3,3,3	0.54	0	2,2,2	0.46	0
6	EDO	S	4	-	3,3,3	0.57	0	2,2,2	0.32	0
6	EDO	S	5	-	3,3,3	0.43	0	2,2,2	0.49	0
7	PGE	S	10	-	9,9,9	0.33	0	8,8,8	0.36	0
6	EDO	S	1	-	3,3,3	0.44	0	2,2,2	0.47	0
6	EDO	S	8	-	3,3,3	0.49	0	2,2,2	0.44	0
6	EDO	S	6	-	3,3,3	0.57	0	2,2,2	0.09	0

In the following table, the Chirals column lists the number of chiral outliers, the number of chiral centers analysed, the number of these observed in the model and the number defined in the Chemical Component Dictionary. Similar counts are reported in the Torsion and Rings columns. '-' means no outliers of that kind were identified.

Mol	Type	Chain	Res	Link	Chirals	Torsions	Rings
6	EDO	S	2	-	-	1/1/1/1	-
6	EDO	S	7	-	-	1/1/1/1	-
7	PGE	S	9	-	-	3/7/7/7	-
6	EDO	S	3	-	-	1/1/1/1	-
6	EDO	S	4	-	-	1/1/1/1	-
6	EDO	S	5	-	-	1/1/1/1	-
7	PGE	S	10	-	-	4/7/7/7	-

Continued on next page...

Continued from previous page...

Mol	Type	Chain	Res	Link	Chirals	Torsions	Rings
6	EDO	S	1	-	-	0/1/1/1	-
6	EDO	S	8	-	-	1/1/1/1	-
6	EDO	S	6	-	-	0/1/1/1	-

There are no bond length outliers.

There are no bond angle outliers.

There are no chirality outliers.

All (13) torsion outliers are listed below:

Mol	Chain	Res	Type	Atoms
7	S	10	PGE	O2-C3-C4-O3
7	S	9	PGE	O3-C5-C6-O4
6	S	8	EDO	O1-C1-C2-O2
7	S	10	PGE	O1-C1-C2-O2
6	S	7	EDO	O1-C1-C2-O2
6	S	5	EDO	O1-C1-C2-O2
7	S	10	PGE	C6-C5-O3-C4
7	S	10	PGE	O3-C5-C6-O4
7	S	9	PGE	O1-C1-C2-O2
6	S	3	EDO	O1-C1-C2-O2
6	S	4	EDO	O1-C1-C2-O2
6	S	2	EDO	O1-C1-C2-O2
7	S	9	PGE	C4-C3-O2-C2

There are no ring outliers.

3 monomers are involved in 6 short contacts:

Mol	Chain	Res	Type	Clashes	Symm-Clashes
6	S	7	EDO	1	0
7	S	9	PGE	1	0
6	S	5	EDO	4	0

The following is a two-dimensional graphical depiction of Mogul quality analysis of bond lengths, bond angles, torsion angles, and ring geometry for all instances of the Ligand of Interest. In addition, ligands with molecular weight > 250 and outliers as shown on the validation Tables will also be included. For torsion angles, if less then 5% of the Mogul distribution of torsion angles is within 10 degrees of the torsion angle in question, then that torsion angle is considered an outlier. Any bond that is central to one or more torsion angles identified as an outlier by Mogul will be highlighted in the graph. For rings, the root-mean-square deviation (RMSD) between the ring in question and similar rings identified by Mogul is calculated over all ring torsion angles. If the average RMSD is greater than 60 degrees and the minimal RMSD between the ring in question and

PRELIMINARY VALIDATION REPORT

any Mogul-identified rings is also greater than 60 degrees, then that ring is considered an outlier. The outliers are highlighted in purple. The color gray indicates Mogul did not find sufficient equivalents in the CSD to analyse the geometry.

Ligand DHP C 2001

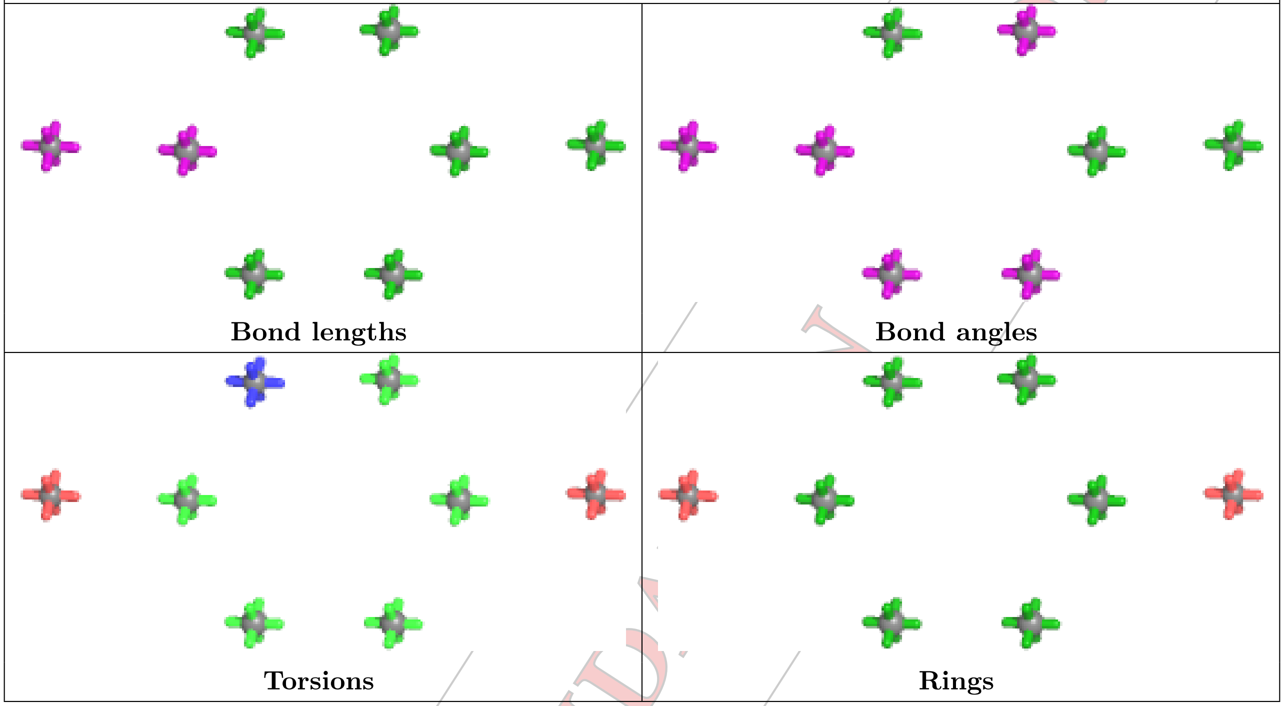

Ligand DHP D 2001

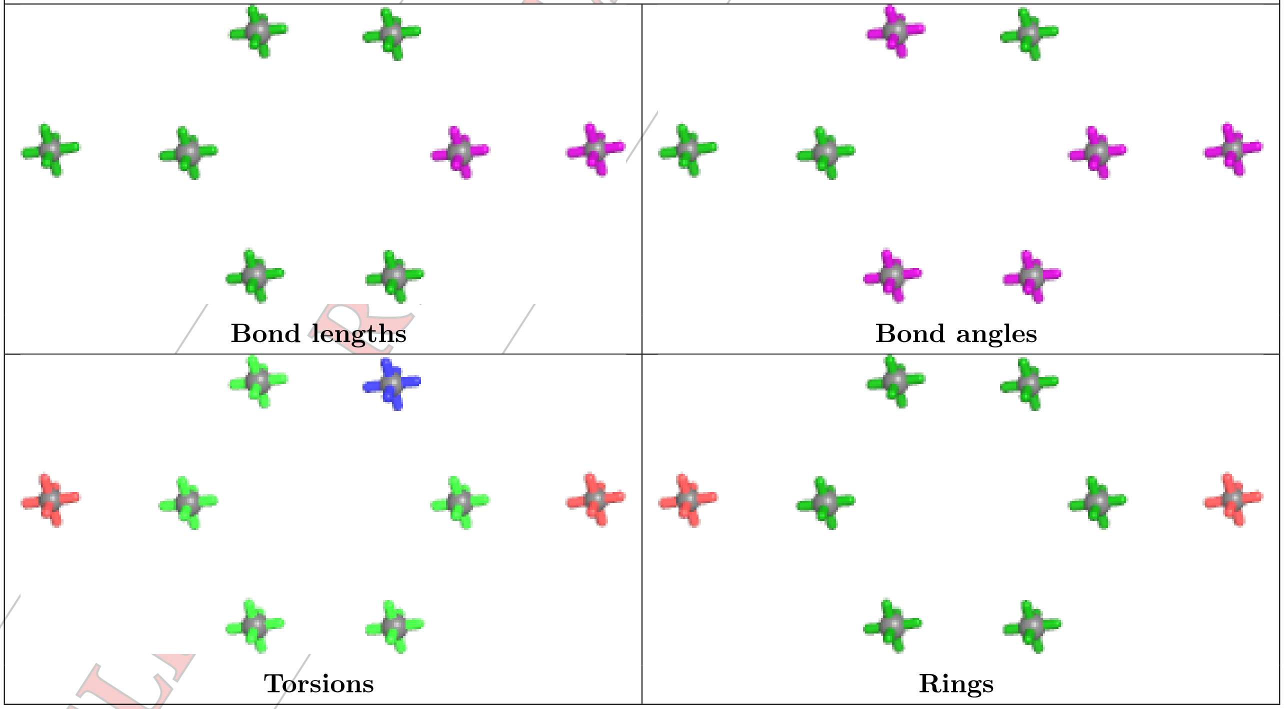

Ligand DHP E 2001

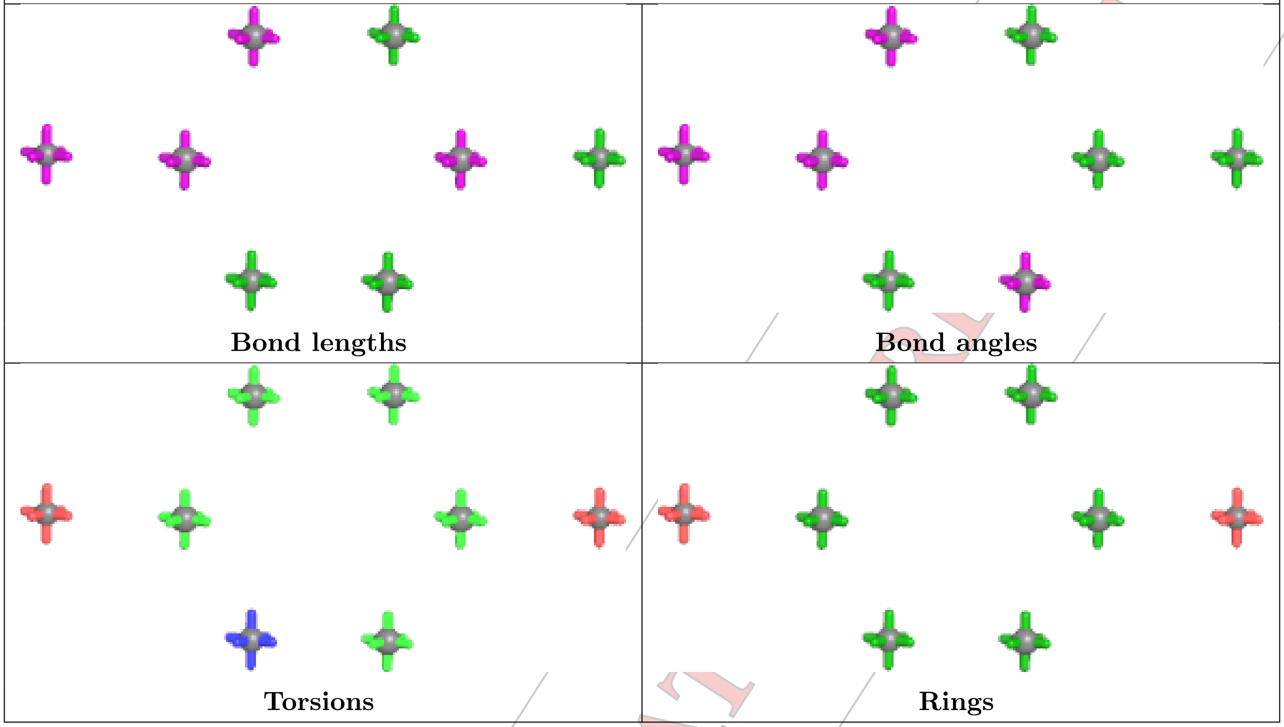

Ligand DHP F 2001

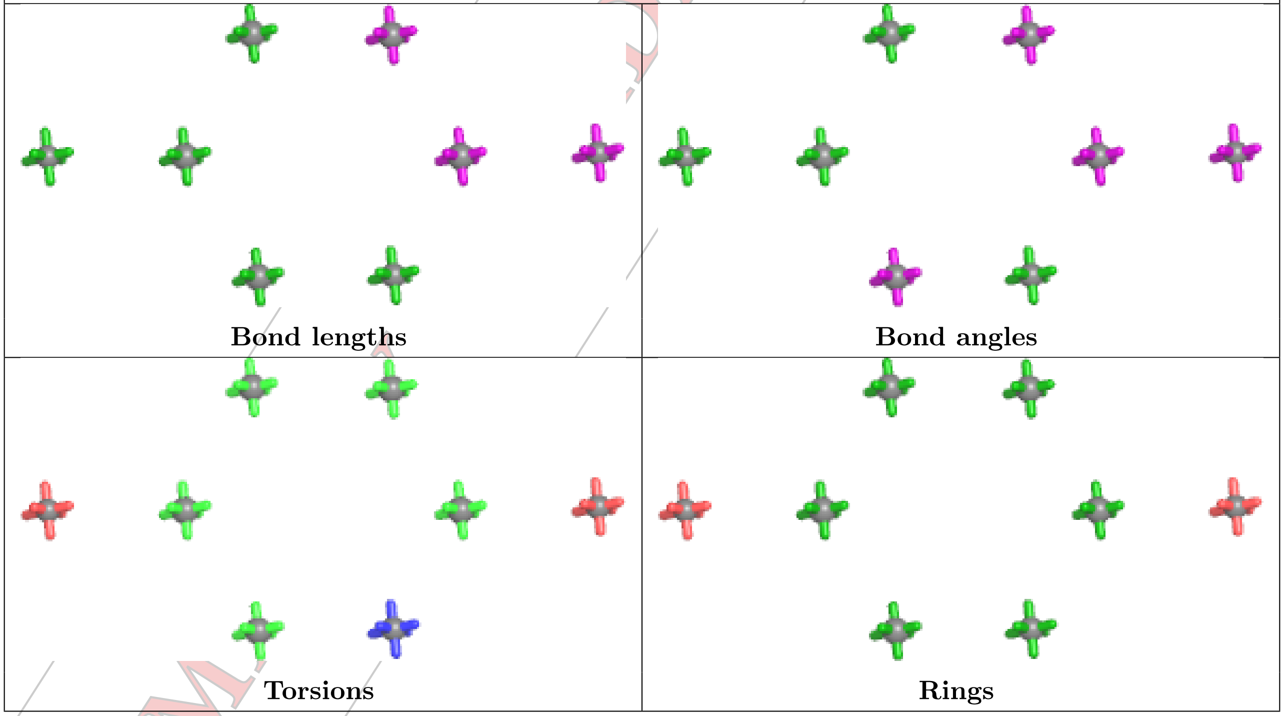

5.7 Other polymers (i)

There are no such residues in this entry.

5.8 Polymer linkage issues (i)

There are no chain breaks in this entry.

PRELIMINARY VALIDATION REPORT

6 Fit of model and data (i)

6.1 Protein, DNA and RNA chains (i)

In the following table, the column labelled '#RSRZ> 2' contains the number (and percentage) of RSRZ outliers, followed by percent RSRZ outliers for the chain as percentile scores relative to all X-ray entries and entries of similar resolution. The OWAB column contains the minimum, median, 95th percentile and maximum values of the occupancy-weighted average B-factor per residue. The column labelled 'Q< 0.9' lists the number of (and percentage) of residues with an average occupancy less than 0.9.

Mol	Chain	Analysed	<RSRZ>	#RSRZ>2			OWAB(Å^2)	Q<0.9
1	A	348/348 (100%)	-0.39	3 (0%)	84	83	20, 32, 49, 64	0
1	B	348/348 (100%)	-0.16	6 (1%)	70	68	23, 37, 57, 72	0
1	E	348/348 (100%)	-0.15	12 (3%)	45	43	19, 34, 57, 69	0
1	F	348/348 (100%)	-0.17	9 (2%)	56	53	20, 34, 54, 66	0
2	C	349/349 (100%)	-0.40	4 (1%)	80	79	19, 29, 50, 76	0
2	D	349/349 (100%)	-0.23	10 (2%)	51	49	22, 34, 55, 82	0
All	All	2090/2090 (100%)	-0.25	44 (2%)	63	61	19, 33, 54, 82	0

All (44) RSRZ outliers are listed below:

Mol	Chain	Res	Type	RSRZ
2	D	350	ARG	4.5
1	E	256	ASN	3.7
1	F	314	LYS	3.7
1	B	300	LEU	3.5
1	E	151	GLN	3.4
1	F	151	GLN	3.4
1	E	153	ARG	3.2
2	C	350	ARG	3.2
2	C	310	GLY	3.2
2	D	311	GLY	3.1
1	B	242	GLY	3.0
1	E	310	GLY	3.0
2	D	312	LYS	2.9
2	D	307	THR	2.8
2	D	332	ASP	2.7
2	C	307	THR	2.7
1	F	300	LEU	2.6
1	B	342	VAL	2.6
1	E	85	LEU	2.6

Continued on next page...

PRELIMINARY VALIDATION REPORT

Continued from previous page...

Mol	Chain	Res	Type	RSRZ
1	E	300	LEU	2.6
1	F	256	ASN	2.6
1	F	150	LYS	2.6
1	F	307	THR	2.5
1	A	153	ARG	2.5
2	D	98	VAL	2.5
1	F	321	ILE	2.4
1	E	309	VAL	2.4
1	E	332	ASP	2.4
1	E	258	PRO	2.4
1	F	266	ILE	2.3
1	B	160	LYS	2.3
1	E	233	LYS	2.3
1	E	321	ILE	2.3
1	E	311	GLY	2.3
2	D	314	LYS	2.3
2	D	310	GLY	2.2
1	A	300	LEU	2.2
1	A	321	ILE	2.2
1	B	310	GLY	2.1
2	C	311	GLY	2.1
1	F	105	HIS	2.0
1	B	153	ARG	2.0
2	D	153	ARG	2.0
2	D	77	GLY	2.0

6.2 Non-standard residues in protein, DNA, RNA chains (i)

There are no non-standard protein/DNA/RNA residues in this entry.

6.3 Carbohydrates (i)

There are no monosaccharides in this entry.

6.4 Ligands (i)

In the following table, the Atoms column lists the number of modelled atoms in the group and the number defined in the chemical component dictionary. The B-factors column lists the minimum, median, 95th percentile and maximum values of B factors of atoms in the group. The column labelled 'Q< 0.9' lists the number of atoms with occupancy less than 0.9.

PRELIMINARY VALIDATION REPORT

Mol	Type	Chain	Res	Atoms	RSCC	RSR	B-factors(Å^2)	Q<0.9
6	EDO	S	6	4/?	0.48	0.31	51,59,61,62	0
7	PGE	S	9	10/?	0.75	0.21	35,50,60,66	0
7	PGE	S	10	10/?	0.79	0.20	37,54,60,64	0
6	EDO	S	8	4/?	0.80	0.20	51,51,63,64	0
3	FE2	C	501	1/?	0.80	0.18	43,43,43,43	0
3	FE2	B	501	1/?	0.83	0.12	44,44,44,44	0
3	FE2	E	501	1/?	0.85	0.12	48,48,48,48	0
4	NFM	A	2001	10/?	0.86	0.20	27,53,66,72	0
4	NFM	B	2001	10/?	0.86	0.20	32,56,67,70	0
6	EDO	S	7	4/?	0.87	0.19	43,44,46,57	0
6	EDO	S	5	4/?	0.87	0.30	55,56,59,61	0
5	DHP	D	2001	8/?	0.87	0.20	28,38,47,48	0
6	EDO	S	4	4/?	0.88	0.35	45,50,51,56	0
5	DHP	C	2001	8/?	0.89	0.16	29,43,47,48	0
3	FE2	D	501	1/?	0.89	0.12	51,51,51,51	0
3	FE2	F	501	1/?	0.90	0.08	48,48,48,48	0
6	EDO	S	2	4/?	0.91	0.27	40,42,43,50	0
5	DHP	E	2001	8/?	0.91	0.18	31,39,51,61	0
6	EDO	S	3	4/?	0.93	0.28	37,40,42,46	0
6	EDO	S	1	4/?	0.93	0.17	49,52,60,62	0
5	DHP	F	2001	8/?	0.95	0.15	31,40,54,68	0
3	FE2	A	501	1/?	0.96	0.10	36,36,36,36	0

The following is a graphical depiction of the model fit to experimental electron density of all instances of the Ligand of Interest. In addition, ligands with molecular weight > 250 and outliers as shown on the geometry validation Tables will also be included. Each fit is shown from different orientation to approximate a three-dimensional view.

PRELIMINARY VALIDATION REPORT

Electron density around DHP D 2001:

$2mF_o$-DF_c (at 0.7 rmsd) in gray
mF_o-DF_c (at 3 rmsd) in purple (negative)
and green (positive)

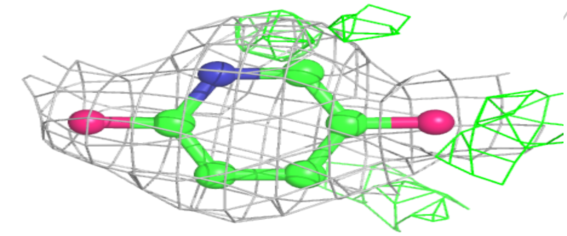

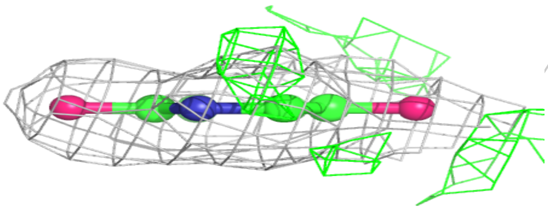

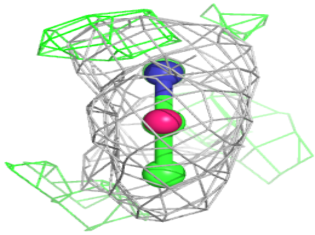

Electron density around DHP C 2001:

$2mF_o$-DF_c (at 0.7 rmsd) in gray
mF_o-DF_c (at 3 rmsd) in purple (negative)
and green (positive)

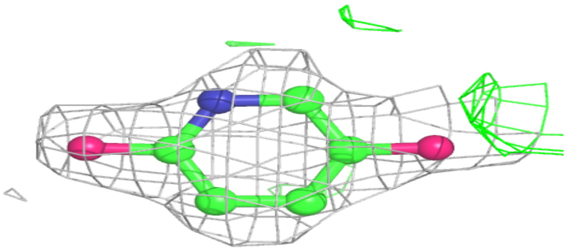

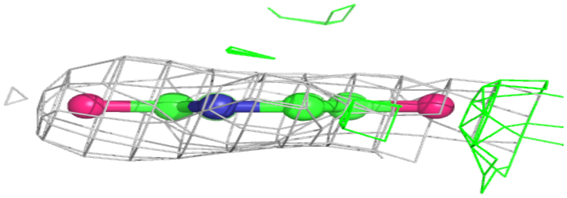

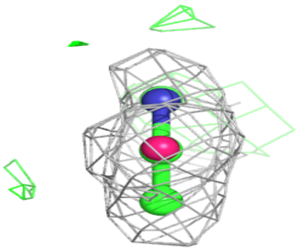

Electron density around DHP E 2001:

$2mF_o$-DF_c (at 0.7 rmsd) in gray
mF_o-DF_c (at 3 rmsd) in purple (negative)
and green (positive)

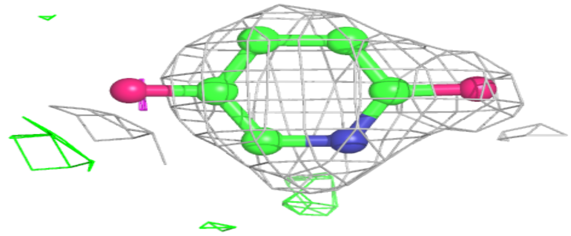

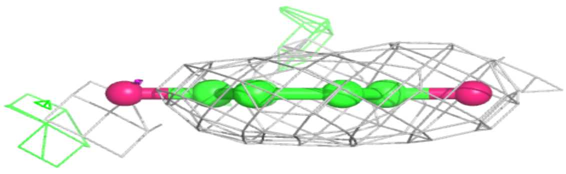

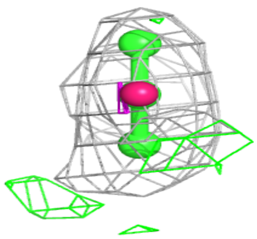

Electron density around DHP F 2001:

$2mF_o$-DF_c (at 0.7 rmsd) in gray
mF_o-DF_c (at 3 rmsd) in purple (negative)
and green (positive)

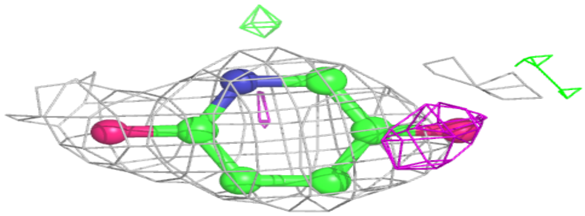

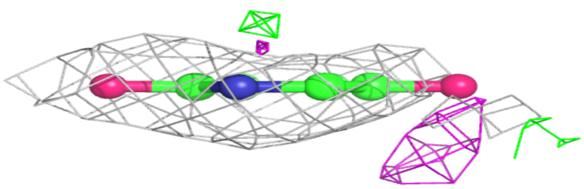

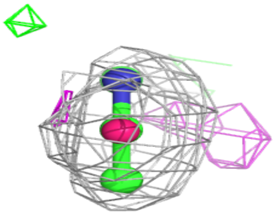

6.5 Other polymers (i)

There are no such residues in this entry.

PRELIMINARY VALIDATION REPORT
