# Supplementary material for: Structure-guided insights into heterocyclic ring-cleavage catalysis of the non-heme Fe (II) dioxygenase NicX
Source: Nat Commun. 2021 Feb 26;12:1301. doi: 10.1038/s41467-021-21567-9 (PMC7910607; doi:10.1038/s41467-021-21567-9)
Supplement: Supplementary file 6 — Source Data [file 41467_2021_21567_MOESM6_ESM.zip › Source data/Source data 2-D_1300017955_val-report-full_P1-7CNT.pdf]

Preliminary Full wwPDB X-ray Structure Validation Report (i)

Aug 3, 2020 – 11:16 AM JST

Deposition ID : D_1300017955

This is a Preliminary Full wwPDB X-ray Structure Validation Report.

This report is produced by the wwPDB Deposition System during initial deposition but before annotation of the structure.

We welcome your comments at *validation@mail.wwpdb.org*
A user guide is available at
https://www.wwpdb.org/validation/2017/XrayValidationReportHelp
with specific help available everywhere you see the (i) symbol.

PRELIMINARY VALIDATION REPORT

The following versions of software and data (see references (i)) were used in the production of this report:

MolProbity	:	4.02b-467
Mogul	:	1.8.5 (274361), CSD as541be (2020)
Xtriage (Phenix)	:	1.13
EDS	:	2.13.1
Percentile statistics	:	20191225.v01 (using entries in the PDB archive December 25th 2019)
Refmac	:	5.8.0158
CCP4	:	7.0.044 (Gargrove)
Ideal geometry (proteins)	:	Engh & Huber (2001)
Ideal geometry (DNA, RNA)	:	Parkinson et al. (1996)
Validation Pipeline (wwPDB-VP)	:	2.13.1

1 Overall quality at a glance (i)

The following experimental techniques were used to determine the structure:
X-RAY DIFFRACTION

The reported resolution of this entry is 2.28 Å.

Percentile scores (ranging between 0-100) for global validation metrics of the entry are shown in the following graphic. The table shows the number of entries on which the scores are based.

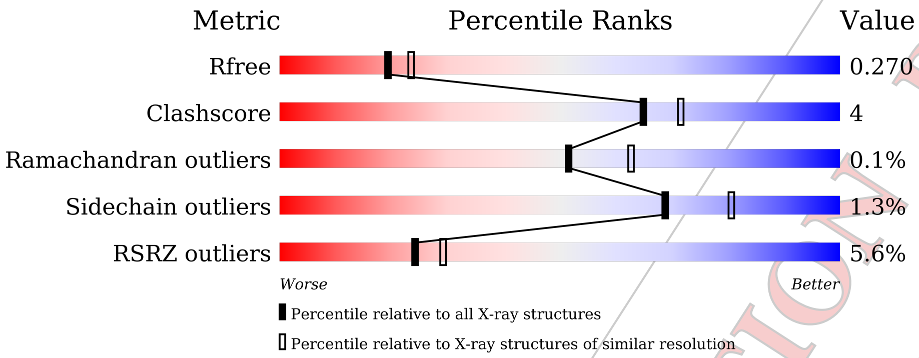

Metric	Whole archive (#Entries)	Similar resolution (#Entries, resolution range(Å))
R_{free}	130704	6980 (2.30-2.26)
Clashscore	141614	7711 (2.30-2.26)
Ramachandran outliers	138981	7597 (2.30-2.26)
Sidechain outliers	138945	7598 (2.30-2.26)
RSRZ outliers	127900	6849 (2.30-2.26)

The table below summarises the geometric issues observed across the polymeric chains and their fit to the electron density. The red, orange, yellow and green segments on the lower bar indicate the fraction of residues that contain outliers for >=3, 2, 1 and 0 types of geometric quality criteria respectively. A grey segment represents the fraction of residues that are not modelled. The numeric value for each fraction is indicated below the corresponding segment, with a dot representing fractions <=5% The upper red bar (where present) indicates the fraction of residues that have poor fit to the electron density. The numeric value is given above the bar.

Mol	Chain	Length	Quality of chain
1	A	348	5% / 89% 11%
1	B	348	5% / 88% 12%
1	C	348	4% / 88% 12%
1	D	348	6% / 93% 6% •
1	E	348	7% / 90% 10%
1	F	348	5% / 91% 9% •

PRELIMINARY VALIDATION REPORT

2 Entry composition (i)

There are 3 unique types of molecules in this entry. The entry contains 16612 atoms, of which 0 are hydrogens and 0 are deuteriums.

In the tables below, the ZeroOcc column contains the number of atoms modelled with zero occupancy, the AltConf column contains the number of residues with at least one atom in alternate conformation and the Trace column contains the number of residues modelled with at most 2 atoms.

- Molecule 1 is a protein.

Mol	Chain	Residues	Atoms						ZeroOcc	AltConf	Trace
			Total	C	N	O	S	Se			
1	A	348	2734	1726	476	516	5	11	0	1	0
1	B	348	2731	1725	473	516	5	12	0	1	0
1	C	348	2723	1720	472	515	5	11	0	0	0
1	D	348	2731	1724	474	517	5	11	0	1	0
1	E	348	2723	1720	472	515	5	11	0	0	0
1	F	348	2723	1720	472	515	5	11	0	0	0

- Molecule 2 is FE (II) ION (three-letter code: FE2) (formula: Fe).

Mol	Chain	Residues	Atoms		ZeroOcc	AltConf
			Total	Fe		
2	A	1	1	1	0	0
2	B	1	1	1	0	0
2	C	1	1	1	0	0
2	D	1	1	1	0	0
2	E	1	1	1	0	0
2	F	1	1	1	0	0

- Molecule 3 is water.

PRELIMINARY VALIDATION REPORT

Mol	Chain	Residues	Atoms		ZeroOcc	AltConf
			Total	O		
3	W	241	241	241	0	0

PRELIMINARY VALIDATION REPORT

3 Residue-property plots (i)

These plots are drawn for all protein, RNA, DNA and oligosaccharide chains in the entry. The first graphic for a chain summarises the proportions of the various outlier classes displayed in the second graphic. The second graphic shows the sequence view annotated by issues in geometry and electron density. Residues are color-coded according to the number of geometric quality criteria for which they contain at least one outlier: green = 0, yellow = 1, orange = 2 and red = 3 or more. A red dot above a residue indicates a poor fit to the electron density (RSRZ > 2). Stretches of 2 or more consecutive residues without any outlier are shown as a green connector. Residues present in the sample, but not in the model, are shown in grey.

- Molecule 1:

Chain A:

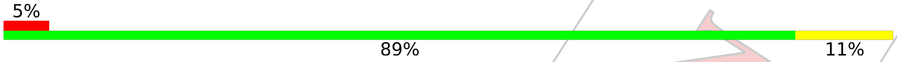

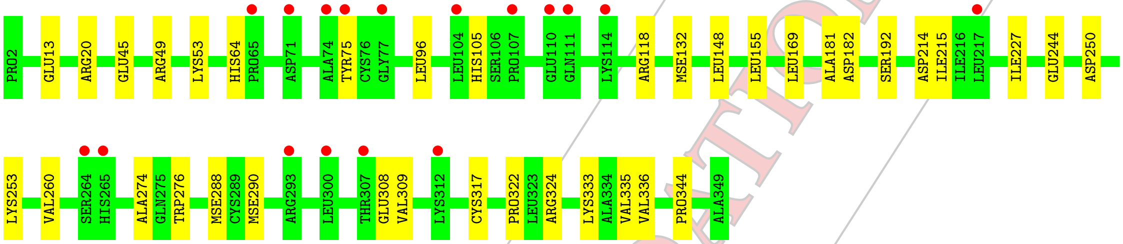

- Molecule 1:

Chain B:

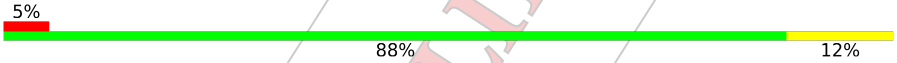

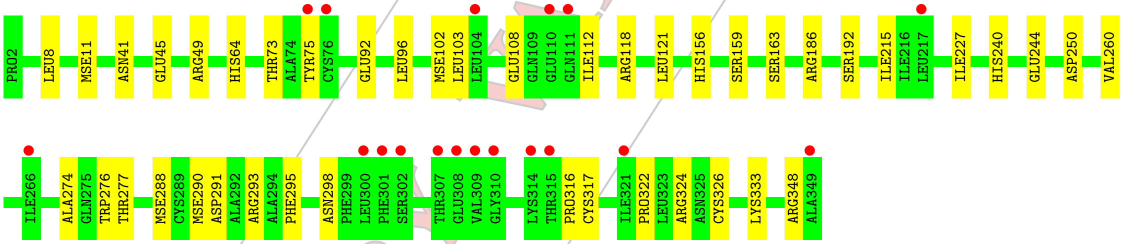

- Molecule 1:

Chain C:

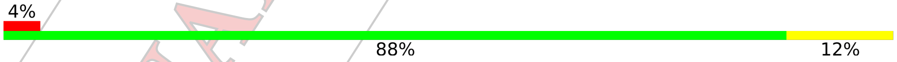

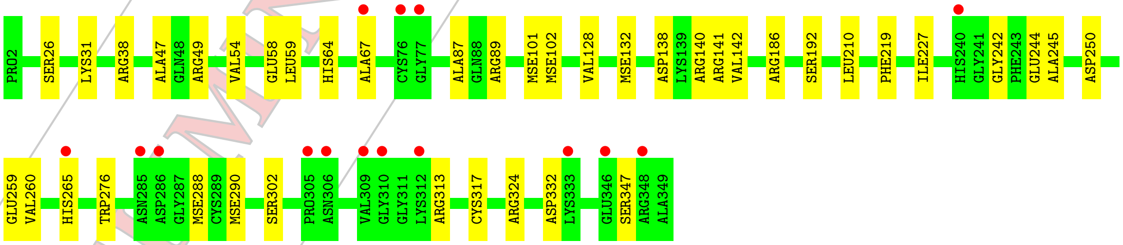

- Molecule 1:

Chain D:

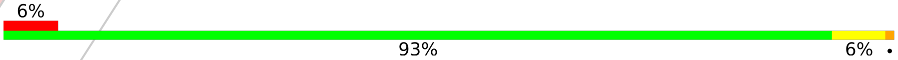

PRELIMINARY VALIDATION REPORT

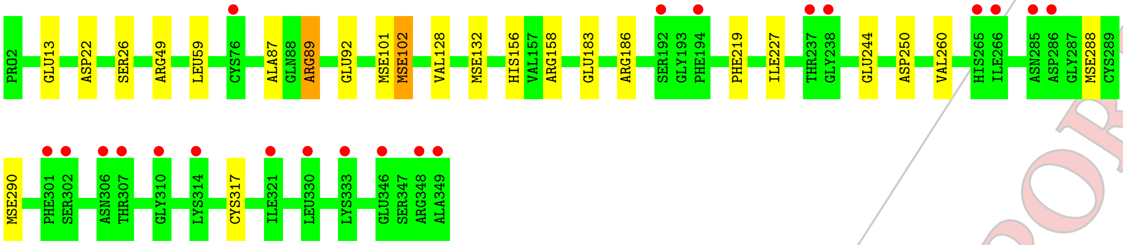

• Molecule 1:

Chain E:

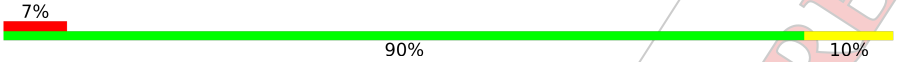

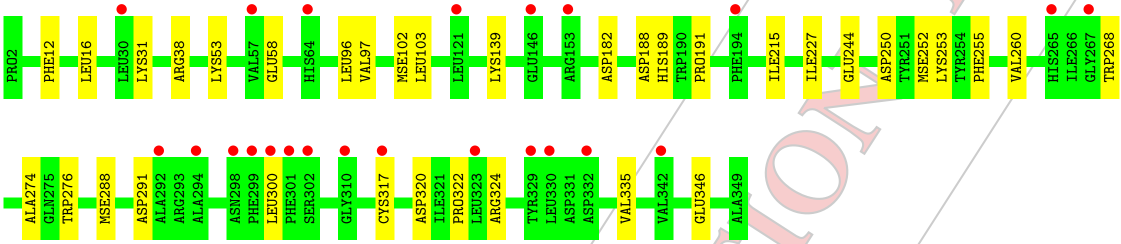

• Molecule 1:

Chain F:

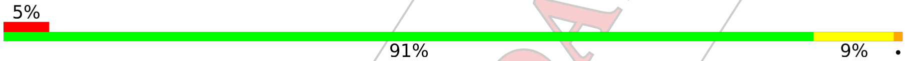

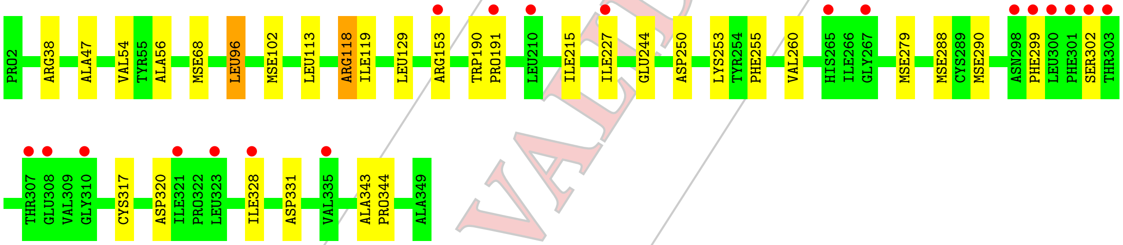

PRELIMINARY VALIDATION REPORT

4 Data and refinement statistics (i)

Property	Value	Source
Space group	P 2 21 21	Depositor
Cell constants<br>a, b, c, α, β, γ	118.89Å 125.92Å 144.13Å<br>90.00° 90.00° 90.00°	Depositor
Resolution (Å)	30.00 – 2.28<br>29.93 – 2.28	Depositor<br>EDS
% Data completeness<br>(in resolution range)	99.0 (30.00-2.28)<br>99.0 (29.93-2.28)	Depositor<br>EDS
R_{merge}	(Not available)	Depositor
R_{sym}	(Not available)	Depositor
$< I/\sigma(I) >$ [1]	2.80 (at 2.29Å)	Xtriage
Refinement program	REFMAC 5.7.0032	Depositor
R, R_{free}	0.211 , 0.267<br>0.220 , 0.270	Depositor<br>DCC
R_{free} test set	4900 reflections (5.00%)	wwPDB-VP
Wilson B-factor (Å^2)	52.6	Xtriage
Anisotropy	0.157	Xtriage
Bulk solvent k_{sol}(e/Å^3), B_{sol}(Å^2)	0.27 , 32.1	EDS
L-test for twinning[2]	$< \|L\| >$ = 0.51, $< L^2 >$ = 0.34	Xtriage
Estimated twinning fraction	No twinning to report.	Xtriage
F_o,F_c correlation	0.96	EDS
Total number of atoms	16612	wwPDB-VP
Average B, all atoms (Å^2)	67.0	wwPDB-VP

Xtriage's analysis on translational NCS is as follows: *The analyses of the Patterson function reveals a significant off-origin peak that is 49.86 % of the origin peak, indicating pseudo-translational symmetry. The chance of finding a peak of this or larger height randomly in a structure without pseudo-translational symmetry is equal to 7.0282e-05. The detected translational NCS is most likely also responsible for the elevated intensity ratio.*

[1] Intensities estimated from amplitudes.

[2] Theoretical values of $< |L| >$, $< L^2 >$ for acentric reflections are 0.5, 0.333 respectively for untwinned datasets, and 0.375, 0.2 for perfectly twinned datasets.

PRELIMINARY VALIDATION REPORT

5 Model quality (i)

5.1 Standard geometry (i)

Bond lengths and bond angles in the following residue types are not validated in this section: FE2

The Z score for a bond length (or angle) is the number of standard deviations the observed value is removed from the expected value. A bond length (or angle) with $|Z| > 5$ is considered an outlier worth inspection. RMSZ is the root-mean-square of all Z scores of the bond lengths (or angles).

Mol	Chain	Bond lengths		Bond angles	
		RMSZ	#|Z| >5	RMSZ	#|Z| >5
1	A	0.71	0/2788	0.85	1/3769 (0.0%)
1	B	0.76	0/2785	0.86	1/3765 (0.0%)
1	C	0.69	0/2777	0.83	3/3755 (0.1%)
1	D	0.69	0/2785	0.82	1/3766 (0.0%)
1	E	0.67	0/2777	0.83	2/3755 (0.1%)
1	F	0.72	0/2777	0.84	3/3755 (0.1%)
All	All	0.71	0/16689	0.84	11/22565 (0.0%)

There are no bond length outliers.

All (11) bond angle outliers are listed below:

Mol	Chain	Res	Type	Atoms	Z	Observed(^{o})	Ideal(^{o})
1	C	141	ARG	NE-CZ-NH1	6.40	123.50	120.30
1	F	38	ARG	NE-CZ-NH2	-6.27	117.16	120.30
1	F	96	LEU	CA-CB-CG	6.24	129.66	115.30
1	E	38	ARG	NE-CZ-NH1	6.00	123.30	120.30
1	A	214	ASP	CB-CG-OD1	5.85	123.57	118.30
1	E	252	MSE	CA-CB-CG	-5.48	103.98	113.30
1	B	348	ARG	NE-CZ-NH1	5.36	122.98	120.30
1	D	89	ARG	NE-CZ-NH1	5.34	122.97	120.30
1	C	89	ARG	NE-CZ-NH1	5.24	122.92	120.30
1	C	141	ARG	NE-CZ-NH2	-5.19	117.70	120.30
1	F	118	ARG	NE-CZ-NH1	5.13	122.87	120.30

There are no chirality outliers.

There are no planarity outliers.

PRELIMINARY VALIDATION REPORT

5.2 Too-close contacts (i)

In the following table, the Non-H and H(model) columns list the number of non-hydrogen atoms and hydrogen atoms in the chain respectively. The H(added) column lists the number of hydrogen atoms added and optimized by MolProbity. The Clashes column lists the number of clashes within the asymmetric unit, whereas Symm-Clashes lists symmetry related clashes.

Mol	Chain	Non-H	H(model)	H(added)	Clashes	Symm-Clashes
1	A	2734	0	2665	23	0
1	B	2731	0	2662	35	0
1	C	2723	0	2654	26	0
1	D	2731	0	2659	18	0
1	E	2723	0	2654	17	0
1	F	2723	0	2654	19	0
2	A	1	0	0	0	0
2	B	1	0	0	0	0
2	C	1	0	0	0	0
2	D	1	0	0	0	0
2	E	1	0	0	0	0
2	F	1	0	0	0	0
3	W	241	0	0	12	0
All	All	16612	0	15948	131	0

The all-atom clashscore is defined as the number of clashes found per 1000 atoms (including hydrogen atoms). The all-atom clashscore for this structure is 4.

All (131) close contacts within the same asymmetric unit are listed below, sorted by their clash magnitude.

Atom-1	Atom-2	Interatomic distance (Å)	Clash overlap (Å)
1:C:102:MSE:HE1	1:C:288:MSE:SE	1.91	1.19
1:E:102:MSE:HE1	1:E:288:MSE:SE	2.03	1.08
1:F:102:MSE:HE1	1:F:288:MSE:SE	2.05	1.06
1:A:288:MSE:HE2	1:A:290:MSE:SE	2.07	1.05
1:B:102[A]:MSE:O	1:B:102[A]:MSE:HG3	1.60	1.01
1:C:102:MSE:CE	1:C:288:MSE:SE	2.63	0.96
1:B:288:MSE:HE2	1:B:290:MSE:SE	2.21	0.90
1:D:158:ARG:HD3	3:W:202:HOH:O	1.84	0.77
1:F:153:ARG:HG2	1:F:331:ASP:OD1	1.87	0.75
1:F:227:ILE:HD11	1:F:244:GLU:HB3	1.70	0.74
1:B:186:ARG:HH11	1:B:186:ARG:HG3	1.52	0.73
1:B:102[A]:MSE:HE1	1:B:288:MSE:SE	2.38	0.73
1:D:102:MSE:CE	1:D:288:MSE:SE	2.88	0.71
1:C:38:ARG:HD3	3:W:104:HOH:O	1.91	0.70

Continued on next page...

PRELIMINARY VALIDATION REPORT

Continued from previous page...

Atom-1	Atom-2	Interatomic distance (Å)	Clash overlap (Å)
1:B:227:ILE:HD11	1:B:244:GLU:HB3	1.74	0.69
1:C:227:ILE:HD11	1:C:244:GLU:HB3	1.74	0.68
1:F:102:MSE:HG3	1:F:102:MSE:O	1.92	0.68
1:E:227:ILE:HD11	1:E:244:GLU:HB3	1.77	0.67
1:B:8:LEU:HD12	1:B:11:MSE:HE2	1.77	0.65
1:A:288:MSE:CE	1:A:290:MSE:SE	2.92	0.64
1:B:108:GLU:HG2	3:W:229:HOH:O	1.99	0.62
1:D:227:ILE:HD11	1:D:244:GLU:HB3	1.80	0.62
1:A:20[A]:ARG:CZ	3:W:228:HOH:O	2.49	0.61
1:C:244:GLU:HB2	3:W:200:HOH:O	2.00	0.60
1:B:102[A]:MSE:CE	1:B:288:MSE:SE	2.99	0.60
1:A:132:MSE:SE	1:A:274:ALA:HB2	2.52	0.59
1:B:260:VAL:HG12	1:B:317:CYS:HB3	1.85	0.59
1:A:227:ILE:HD11	1:A:244:GLU:HB3	1.85	0.58
1:A:20[A]:ARG:NH2	3:W:228:HOH:O	2.36	0.58
1:E:53:LYS:HD3	3:W:89:HOH:O	2.03	0.57
1:E:188:ASP:OD1	1:E:189:HIS:N	2.35	0.57
1:B:186:ARG:HG3	1:B:186:ARG:NH1	2.14	0.57
1:B:156:HIS:HB2	3:W:199:HOH:O	2.04	0.57
1:A:96:LEU:HD23	1:A:118:ARG:HG3	1.86	0.56
1:D:156:HIS:CE1	3:W:44:HOH:O	2.57	0.56
1:F:260:VAL:HG12	1:F:317:CYS:HB3	1.87	0.56
1:A:276:TRP:CD1	1:A:324:ARG:HD2	2.42	0.55
1:A:13:GLU:OE2	1:A:49:ARG:NH1	2.40	0.55
1:A:260:VAL:HG12	1:A:317:CYS:HB3	1.89	0.54
1:F:227:ILE:HD11	1:F:244:GLU:CB	2.37	0.54
1:B:92:GLU:HG3	1:B:112:ILE:HG12	1.90	0.53
1:D:102:MSE:HE1	1:D:288:MSE:SE	2.58	0.53
1:D:13:GLU:OE2	1:D:49:ARG:NH1	2.41	0.53
1:D:288:MSE:HE2	1:D:290:MSE:SE	2.59	0.53
1:B:260:VAL:CG1	1:B:317:CYS:HB3	2.39	0.53
1:A:155:LEU:HB2	1:A:169:LEU:HD11	1.92	0.51
1:E:300:LEU:HD11	1:E:320:ASP:HB3	1.93	0.51
1:A:148:LEU:HD11	1:A:333:LYS:NZ	2.26	0.50
1:B:49:ARG:NH1	1:C:49:ARG:HD3	2.26	0.50
1:B:102[A]:MSE:HA	1:B:121:LEU:HD11	1.94	0.50
1:F:47:ALA:HB3	1:F:54:VAL:HG11	1.92	0.50
1:D:13:GLU:OE2	1:D:49:ARG:CZ	2.60	0.49
1:F:113:LEU:HD21	1:F:119:ILE:HD12	1.95	0.49
1:A:335:VAL:HG23	1:A:336:VAL:HG23	1.94	0.49
1:C:242:GLY:O	1:C:245:ALA:HB3	2.13	0.49

Continued on next page...

PRELIMINARY VALIDATION REPORT

Continued from previous page...

Atom-1	Atom-2	Interatomic distance (Å)	Clash overlap (Å)
1:C:265:HIS:CE1	1:C:302:SER:HB2	2.48	0.48
1:B:102[B]:MSE:HA	1:B:121:LEU:HD11	1.95	0.48
1:F:96:LEU:HD23	1:F:118:ARG:HG3	1.96	0.48
1:A:64:HIS:CG	1:A:75:TYR:CD1	3.02	0.48
1:D:128:VAL:HG13	1:D:132:MSE:CE	2.44	0.48
1:A:148:LEU:HD22	1:A:344:PRO:HG3	1.96	0.47
1:F:129:LEU:HB3	1:F:190:TRP:CZ2	2.49	0.47
1:A:118:ARG:HD2	1:A:181:ALA:O	2.13	0.47
1:B:326:CYS:HB2	3:W:121:HOH:O	2.15	0.47
1:B:277:THR:OG1	1:F:68:MSE:HE1	2.15	0.47
1:B:295:PHE:CE2	1:B:298:ASN:HB3	2.50	0.47
1:B:215:ILE:HB	1:B:322:PRO:HG2	1.97	0.47
1:E:268:TRP:CH2	1:E:335:VAL:HG11	2.49	0.47
1:B:159:SER:OG	1:B:163:SER:HB2	2.15	0.47
1:C:47:ALA:HB3	1:C:54:VAL:HG11	1.98	0.46
1:E:96:LEU:HD12	1:E:97:VAL:N	2.31	0.46
1:C:210:LEU:HD22	1:C:227:ILE:HD12	1.97	0.46
1:C:128:VAL:CG1	1:C:132:MSE:HE3	2.46	0.46
1:D:101:MSE:O	1:D:102:MSE:HB3	2.16	0.46
1:F:288:MSE:HE2	1:F:290:MSE:SE	2.66	0.46
1:D:260:VAL:HG12	1:D:317:CYS:HB3	1.98	0.45
1:B:316:PRO:HD2	3:W:230:HOH:O	2.15	0.45
1:B:276:TRP:CD2	1:B:324:ARG:HD2	2.51	0.45
1:A:215:ILE:HB	1:A:322:PRO:HG2	1.98	0.45
1:E:274:ALA:HB1	1:E:291:ASP:HA	1.98	0.45
1:A:276:TRP:CG	1:A:324:ARG:HD2	2.52	0.45
1:C:31:LYS:O	1:C:58:GLU:HA	2.15	0.45
1:E:12:PHE:O	1:E:16:LEU:HG	2.17	0.45
1:A:148:LEU:HA	1:A:148:LEU:HD12	1.81	0.45
1:F:215:ILE:HG23	1:F:279:MSE:HE1	1.98	0.45
1:B:49:ARG:NE	1:C:49:ARG:CZ	2.80	0.45
1:E:31:LYS:O	1:E:58:GLU:HA	2.16	0.44
1:A:49:ARG:HD3	1:D:49:ARG:NH1	2.32	0.44
1:C:259:GLU:HB3	1:C:313:ARG:HD2	2.00	0.44
1:B:240:HIS:ND1	3:W:42:HOH:O	2.36	0.44
1:B:274:ALA:HB1	1:B:291:ASP:HA	1.99	0.44
1:E:102:MSE:HG3	1:E:102:MSE:O	2.17	0.44
1:C:138:ASP:O	1:C:142:VAL:HG23	2.17	0.44
1:F:299:PHE:HB2	1:F:328:ILE:HD11	2.00	0.44
1:E:139:LYS:NZ	1:E:182:ASP:OD2	2.50	0.44
1:E:276:TRP:CD1	1:E:324:ARG:HD2	2.53	0.43

Continued on next page...

PRELIMINARY VALIDATION REPORT

Continued from previous page...

Atom-1	Atom-2	Interatomic distance (Å)	Clash overlap (Å)
1:E:215:ILE:O	1:E:322:PRO:HD2	2.18	0.43
1:C:276:TRP:CE2	1:C:324:ARG:HD2	2.54	0.43
1:B:49:ARG:CZ	1:C:49:ARG:NE	2.81	0.43
1:D:219:PHE:N	1:D:219:PHE:CD1	2.87	0.43
1:E:255:PHE:CD2	1:E:317:CYS:HB2	2.54	0.42
1:E:260:VAL:HG12	1:E:317:CYS:HB3	2.00	0.42
1:C:64:HIS:HB2	1:C:67:ALA:HB2	2.01	0.42
1:C:59:LEU:HD21	1:C:87:ALA:HB1	2.01	0.42
1:B:49:ARG:HD3	1:C:49:ARG:NH1	2.35	0.42
1:F:255:PHE:CG	1:F:317:CYS:HB2	2.55	0.42
1:F:343:ALA:HA	1:F:344:PRO:C	2.39	0.42
1:A:105:HIS:HB3	1:A:308:GLU:HG2	2.02	0.41
1:A:20[A]:ARG:NH1	1:A:182:ASP:O	2.54	0.41
1:B:186:ARG:NH1	1:B:186:ARG:CG	2.80	0.41
1:B:73:THR:HG23	1:B:102[A]:MSE:HE3	2.02	0.41
1:D:22:ASP:OD1	1:D:22:ASP:C	2.59	0.41
1:D:89:ARG:NH1	1:D:92:GLU:OE1	2.53	0.41
1:F:190:TRP:HA	1:F:191:PRO:HA	1.92	0.41
1:B:41:ASN:O	1:B:45:GLU:HG2	2.20	0.41
1:D:59:LEU:HD21	1:D:87:ALA:HB1	2.03	0.41
1:D:183:GLU:O	1:D:186:ARG:HB3	2.21	0.41
1:C:288:MSE:HE2	1:C:290:MSE:SE	2.70	0.41
1:C:260:VAL:HG12	1:C:317:CYS:HB3	2.01	0.41
1:B:96:LEU:HD12	1:B:118:ARG:HG3	2.01	0.41
1:B:64:HIS:CG	1:B:75:TYR:CD1	3.08	0.41
1:A:260:VAL:CG1	1:A:317:CYS:HB3	2.50	0.41
1:C:276:TRP:NE1	1:C:324:ARG:HD2	2.36	0.41
1:F:302:SER:HB2	1:F:320:ASP:OD1	2.21	0.41
1:C:101:MSE:O	1:C:102:MSE:HB3	2.20	0.40
1:B:103:LEU:HA	1:B:103:LEU:HD23	1.98	0.40
1:D:128:VAL:HG13	1:D:132:MSE:HE3	2.04	0.40
1:B:290:MSE:SE	1:B:293:ARG:HD2	2.71	0.40
1:C:219:PHE:CD1	1:C:219:PHE:N	2.88	0.40
1:E:103:LEU:HA	1:E:103:LEU:HD23	1.97	0.40
1:C:58:GLU:HB3	1:F:56:ALA:HB3	2.04	0.40

There are no symmetry-related clashes.

PRELIMINARY VALIDATION REPORT

5.3 Torsion angles (i)

5.3.1 Protein backbone (i)

In the following table, the Percentiles column shows the percent Ramachandran outliers of the chain as a percentile score with respect to all X-ray entries followed by that with respect to entries of similar resolution.

The Analysed column shows the number of residues for which the backbone conformation was analysed, and the total number of residues.

Mol	Chain	Analysed	Favoured	Allowed	Outliers	Percentiles	
1	A	347/348 (100%)	330 (95%)	16 (5%)	1 (0%)	41	49
1	B	347/348 (100%)	328 (94%)	18 (5%)	1 (0%)	41	49
1	C	346/348 (99%)	333 (96%)	12 (4%)	1 (0%)	41	49
1	D	347/348 (100%)	333 (96%)	14 (4%)	0	100	100
1	E	346/348 (99%)	324 (94%)	22 (6%)	0	100	100
1	F	346/348 (99%)	328 (95%)	18 (5%)	0	100	100
All	All	2079/2088 (100%)	1976 (95%)	100 (5%)	3 (0%)	51	63

All (3) Ramachandran outliers are listed below:

Mol	Chain	Res	Type
1	B	192	SER
1	A	192	SER
1	C	192	SER

5.3.2 Protein sidechains (i)

In the following table, the Percentiles column shows the percent sidechain outliers of the chain as a percentile score with respect to all X-ray entries followed by that with respect to entries of similar resolution.

The Analysed column shows the number of residues for which the sidechain conformation was analysed, and the total number of residues.

Mol	Chain	Analysed	Rotameric	Outliers	Percentiles	
1	A	288/276 (104%)	283 (98%)	5 (2%)	60	74
1	B	288/276 (104%)	286 (99%)	2 (1%)	84	91
1	C	287/276 (104%)	281 (98%)	6 (2%)	53	68
1	D	288/276 (104%)	285 (99%)	3 (1%)	76	86

Continued on next page...

PRELIMINARY VALIDATION REPORT

Continued from previous page...

Mol	Chain	Analysed	Rotameric	Outliers	Percentiles	
1	E	287/276 (104%)	283 (99%)	4 (1%)	67	79
1	F	287/276 (104%)	285 (99%)	2 (1%)	84	91
All	All	1725/1656 (104%)	1703 (99%)	22 (1%)	69	80

All (22) residues with a non-rotameric sidechain are listed below:

Mol	Chain	Res	Type
1	A	45	GLU
1	A	53	LYS
1	A	250	ASP
1	A	253	LYS
1	A	309	VAL
1	B	250	ASP
1	B	333	LYS
1	C	26	SER
1	C	140	ARG
1	C	186	ARG
1	C	250	ASP
1	C	332	ASP
1	C	347	SER
1	D	26	SER
1	D	102	MSE
1	D	250	ASP
1	E	191	PRO
1	E	250	ASP
1	E	253	LYS
1	E	346	GLU
1	F	250	ASP
1	F	253	LYS

Some sidechains can be flipped to improve hydrogen bonding and reduce clashes. All (9) such sidechains are listed below:

Mol	Chain	Res	Type
1	A	151	GLN
1	A	189	HIS
1	B	70	ASN
1	B	189	HIS
1	B	282	HIS
1	B	285	ASN
1	C	189	HIS

Continued on next page...

PRELIMINARY VALIDATION REPORT

Continued from previous page...

Mol	Chain	Res	Type
1	C	265	HIS
1	F	265	HIS

5.3.3 RNA (i)

There are no RNA molecules in this entry.

5.4 Non-standard residues in protein, DNA, RNA chains (i)

There are no non-standard protein/DNA/RNA residues in this entry.

5.5 Carbohydrates (i)

There are no monosaccharides in this entry.

5.6 Ligand geometry (i)

Of 6 ligands modelled in this entry, 6 are monoatomic - leaving 0 for Mogul analysis.

There are no bond length outliers.

There are no bond angle outliers.

There are no chirality outliers.

There are no torsion outliers.

There are no ring outliers.

No monomer is involved in short contacts.

5.7 Other polymers (i)

There are no such residues in this entry.

5.8 Polymer linkage issues (i)

There are no chain breaks in this entry.

PRELIMINARY VALIDATION REPORT

6 Fit of model and data (i)

6.1 Protein, DNA and RNA chains (i)

In the following table, the column labelled '#RSRZ> 2' contains the number (and percentage) of RSRZ outliers, followed by percent RSRZ outliers for the chain as percentile scores relative to all X-ray entries and entries of similar resolution. The OWAB column contains the minimum, median, 95th percentile and maximum values of the occupancy-weighted average B-factor per residue. The column labelled 'Q< 0.9' lists the number of (and percentage) of residues with an average occupancy less than 0.9.

Mol	Chain	Analysed	<RSRZ>	#RSRZ>2			OWAB(Å^2)	Q<0.9
1	A	337/348 (96%)	0.16	17 (5%)	28	34	45, 62, 105, 131	0
1	B	337/348 (96%)	0.09	18 (5%)	26	31	40, 57, 102, 145	0
1	C	337/348 (96%)	0.05	15 (4%)	33	39	39, 62, 103, 127	0
1	D	337/348 (96%)	0.09	21 (6%)	20	25	44, 63, 100, 128	0
1	E	337/348 (96%)	0.21	23 (6%)	17	21	45, 69, 104, 139	0
1	F	337/348 (96%)	0.07	19 (5%)	24	29	39, 60, 90, 120	0
All	All	2022/2088 (96%)	0.11	113 (5%)	24	29	39, 62, 101, 145	0

All (113) RSRZ outliers are listed below:

Mol	Chain	Res	Type	RSRZ
1	E	310	GLY	5.8
1	F	300	LEU	5.7
1	E	300	LEU	5.4
1	D	348	ARG	5.1
1	A	75	TYR	4.7
1	E	329	TYR	4.4
1	B	307	THR	4.3
1	B	75	TYR	4.3
1	D	76	CYS	4.3
1	A	110	GLU	4.2
1	E	265	HIS	4.0
1	A	107	PRO	4.0
1	B	309	VAL	3.9
1	B	308	GLU	3.7
1	C	310	GLY	3.7
1	F	301	PHE	3.6
1	F	328	ILE	3.6
1	F	307	THR	3.6
1	D	265	HIS	3.6

Continued on next page...

PRELIMINARY VALIDATION REPORT

Continued from previous page...

Mol	Chain	Res	Type	RSRZ
1	A	300	LEU	3.6
1	B	301	PHE	3.5
1	F	310	GLY	3.3
1	E	194	PHE	3.3
1	B	76	CYS	3.3
1	D	310	GLY	3.3
1	F	335	VAL	3.2
1	A	111	GLN	3.2
1	A	265	HIS	3.2
1	A	65	PRO	3.2
1	D	330	LEU	3.2
1	F	323	LEU	3.1
1	F	308	GLU	3.1
1	A	114	LYS	3.0
1	D	266	ILE	3.0
1	C	346	GLU	2.9
1	C	240	HIS	2.9
1	E	330	LEU	2.9
1	B	321	ILE	2.9
1	E	342	VAL	2.9
1	A	77	GLY	2.9
1	B	314	LYS	2.8
1	C	77	GLY	2.8
1	E	153	ARG	2.8
1	C	285	ASN	2.8
1	E	301	PHE	2.7
1	F	210	LEU	2.7
1	E	57	VAL	2.7
1	D	314	LYS	2.7
1	C	265	HIS	2.7
1	F	299	PHE	2.7
1	E	121	LEU	2.7
1	D	307	THR	2.7
1	F	321	ILE	2.6
1	B	310	GLY	2.6
1	F	267	GLY	2.6
1	E	302	SER	2.6
1	C	333	LYS	2.6
1	D	333	LYS	2.6
1	C	348	ARG	2.5
1	E	30	LEU	2.5
1	A	71	ASP	2.5

Continued on next page...

PRELIMINARY VALIDATION REPORT

Continued from previous page...

Mol	Chain	Res	Type	RSRZ
1	B	110	GLU	2.5
1	C	305	PRO	2.5
1	D	238	GLY	2.5
1	E	267	GLY	2.5
1	A	264	SER	2.4
1	C	76	CYS	2.4
1	A	74	ALA	2.4
1	D	285	ASN	2.4
1	F	303	THR	2.4
1	D	302	SER	2.4
1	E	323	LEU	2.4
1	E	294	ALA	2.4
1	F	227	ILE	2.4
1	F	265	HIS	2.4
1	C	306	ASN	2.4
1	E	298	ASN	2.4
1	C	286	ASP	2.3
1	C	312	LYS	2.3
1	B	300	LEU	2.3
1	B	315	THR	2.3
1	D	286	ASP	2.3
1	A	312	LYS	2.3
1	E	64	HIS	2.3
1	F	153	ARG	2.3
1	E	317	CYS	2.3
1	B	217	LEU	2.2
1	D	194	PHE	2.2
1	A	217	LEU	2.2
1	A	293	ARG	2.2
1	D	301	PHE	2.2
1	A	307	THR	2.2
1	F	191	PRO	2.2
1	D	306	ASN	2.1
1	B	349	ALA	2.1
1	B	266	ILE	2.1
1	D	192	SER	2.1
1	F	302	SER	2.1
1	C	309	VAL	2.1
1	E	292	ALA	2.1
1	B	302	SER	2.1
1	D	321	ILE	2.1
1	B	111	GLN	2.1

Continued on next page...

PRELIMINARY VALIDATION REPORT

Continued from previous page...

Mol	Chain	Res	Type	RSRZ
1	F	298	ASN	2.1
1	A	104	LEU	2.1
1	E	332	ASP	2.0
1	D	346	GLU	2.0
1	E	146	GLU	2.0
1	B	104	LEU	2.0
1	D	237	THR	2.0
1	C	67	ALA	2.0
1	D	349	ALA	2.0
1	E	299	PHE	2.0

6.2 Non-standard residues in protein, DNA, RNA chains (i)

There are no non-standard protein/DNA/RNA residues in this entry.

6.3 Carbohydrates (i)

There are no monosaccharides in this entry.

6.4 Ligands (i)

In the following table, the Atoms column lists the number of modelled atoms in the group and the number defined in the chemical component dictionary. The B-factors column lists the minimum, median, 95th percentile and maximum values of B factors of atoms in the group. The column labelled 'Q< 0.9' lists the number of atoms with occupancy less than 0.9.

Mol	Type	Chain	Res	Atoms	RSCC	RSR	B-factors($Å^2$)	Q<0.9
2	FE2	B	501	1/?	0.75	0.18	112,112,112,112	0
2	FE2	A	501	1/?	0.92	0.12	110,110,110,110	0
2	FE2	C	501	1/?	0.94	0.18	103,103,103,103	0
2	FE2	D	501	1/?	0.96	0.10	93,93,93,93	0
2	FE2	E	501	1/?	0.98	0.17	89,89,89,89	0
2	FE2	F	501	1/?	0.99	0.09	72,72,72,72	0

6.5 Other polymers (i)

There are no such residues in this entry.

PRELIMINARY VALIDATION REPORT
