# Supplementary material for: Structure-guided insights into heterocyclic ring-cleavage catalysis of the non-heme Fe (II) dioxygenase NicX
Source: Nat Commun. 2021 Feb 26;12:1301. doi: 10.1038/s41467-021-21567-9 (PMC7910607; doi:10.1038/s41467-021-21567-9)
Supplement: Supplementary file 6 — Source Data [file 41467_2021_21567_MOESM6_ESM.zip › Source data/Source data 3-D_1300018285_val-report-full_P1-7CUP.pdf]

Preliminary Full wwPDB X-ray Structure Validation Report (i)

Aug 23, 2020 – 09:50 PM JST

Deposition ID : D_1300018285

This is a Preliminary Full wwPDB X-ray Structure Validation Report.

This report is produced by the wwPDB Deposition System during initial deposition but before annotation of the structure.

We welcome your comments at *validation@mail.wwpdb.org*
A user guide is available at
https://www.wwpdb.org/validation/2017/XrayValidationReportHelp
with specific help available everywhere you see the (i) symbol.

PRELIMINARY VALIDATION REPORT

The following versions of software and data (see references (i)) were used in the production of this report:

MolProbity	:	4.02b-467
Xtriage (Phenix)	:	1.13
EDS	:	2.13.1
Percentile statistics	:	20191225.v01 (using entries in the PDB archive December 25th 2019)
Refmac	:	5.8.0158
CCP4	:	7.0.044 (Gargrove)
Ideal geometry (proteins)	:	Engh & Huber (2001)
Ideal geometry (DNA, RNA)	:	Parkinson et al. (1996)
Validation Pipeline (wwPDB-VP)	:	2.13.1

1 Overall quality at a glance (i)

The following experimental techniques were used to determine the structure:
X-RAY DIFFRACTION

The reported resolution of this entry is 2.00 Å.

Percentile scores (ranging between 0-100) for global validation metrics of the entry are shown in the following graphic. The table shows the number of entries on which the scores are based.

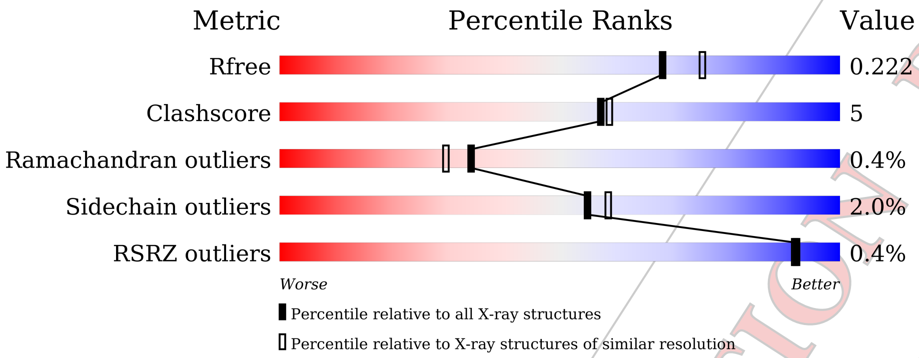

Metric	Whole archive (#Entries)	Similar resolution (#Entries, resolution range(Å))
R_{free}	130704	8085 (2.00-2.00)
Clashscore	141614	9178 (2.00-2.00)
Ramachandran outliers	138981	9054 (2.00-2.00)
Sidechain outliers	138945	9053 (2.00-2.00)
RSRZ outliers	127900	7900 (2.00-2.00)

The table below summarises the geometric issues observed across the polymeric chains and their fit to the electron density. The red, orange, yellow and green segments on the lower bar indicate the fraction of residues that contain outliers for >=3, 2, 1 and 0 types of geometric quality criteria respectively. A grey segment represents the fraction of residues that are not modelled. The numeric value for each fraction is indicated below the corresponding segment, with a dot representing fractions <=5% The upper red bar (where present) indicates the fraction of residues that have poor fit to the electron density. The numeric value is given above the bar.

Mol	Chain	Length	Quality of chain
1	A	348	% 86% 13% •
1	B	348	88% 10% •
1	C	348	91% 8% •
1	D	348	92% 7%
2	E	347	88% 11% •
2	F	347	% 90% 10%

PRELIMINARY VALIDATION REPORT

2 Entry composition (i)

There are 4 unique types of molecules in this entry. The entry contains 17845 atoms, of which 0 are hydrogens and 0 are deuteriums.

In the tables below, the ZeroOcc column contains the number of atoms modelled with zero occupancy, the AltConf column contains the number of residues with at least one atom in alternate conformation and the Trace column contains the number of residues modelled with at most 2 atoms.

- Molecule 1 is a protein.

Mol	Chain	Residues	Atoms	ZeroOcc	AltConf	Trace
1	A	348	Total C N O S<br>2723 1720 472 515 16	0	0	0
1	B	348	Total C N O S<br>2723 1720 472 515 16	0	0	0
1	C	348	Total C N O S<br>2723 1720 472 515 16	0	0	0
1	D	348	Total C N O S<br>2723 1720 472 515 16	0	0	0

- Molecule 2 is a protein.

Mol	Chain	Residues	Atoms	ZeroOcc	AltConf	Trace
2	E	347	Total C N O S<br>2718 1717 471 514 16	0	0	0
2	F	347	Total C N O S<br>2718 1717 471 514 16	0	0	0

- Molecule 3 is FE (III) ION (three-letter code: FE) (formula: Fe).

Mol	Chain	Residues	Atoms	ZeroOcc	AltConf
3	G	1	Total Fe<br>1 1	0	0
3	G	1	Total Fe<br>1 1	0	0
3	G	1	Total Fe<br>1 1	0	0
3	G	1	Total Fe<br>1 1	0	0
3	G	1	Total Fe<br>1 1	0	0
3	G	1	Total Fe<br>1 1	0	0

PRELIMINARY VALIDATION REPORT

- Molecule 4 is water.

Mol	Chain	Residues	Atoms		ZeroOcc	AltConf
4	S	1511	Total<br>1511	O<br>1511	0	0

PRELIMINARY VALIDATION REPORT

3 Residue-property plots (i)

These plots are drawn for all protein, RNA, DNA and oligosaccharide chains in the entry. The first graphic for a chain summarises the proportions of the various outlier classes displayed in the second graphic. The second graphic shows the sequence view annotated by issues in geometry and electron density. Residues are color-coded according to the number of geometric quality criteria for which they contain at least one outlier: green = 0, yellow = 1, orange = 2 and red = 3 or more. A red dot above a residue indicates a poor fit to the electron density (RSRZ > 2). Stretches of 2 or more consecutive residues without any outlier are shown as a green connector. Residues present in the sample, but not in the model, are shown in grey.

- Molecule 1:

Chain A:

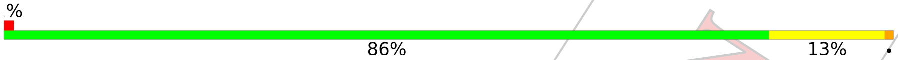

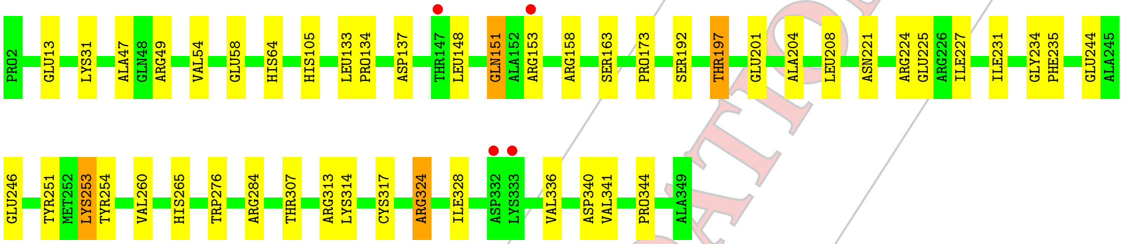

- Molecule 1:

Chain B:

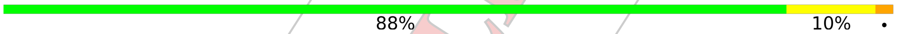

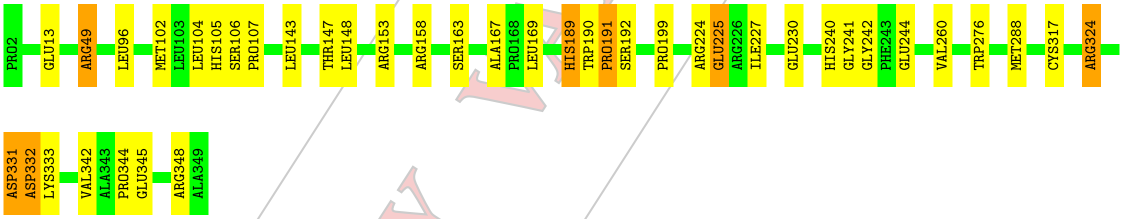

- Molecule 1:

Chain C:

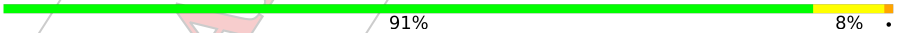

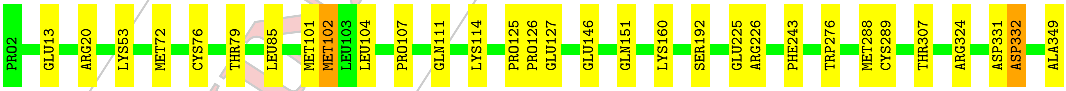

- Molecule 1:

Chain D:

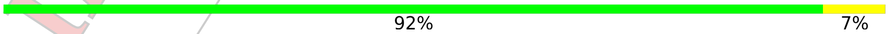

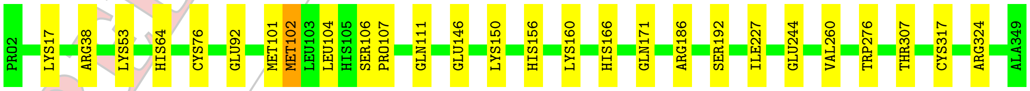

- Molecule 2:

PRELIMINARY VALIDATION REPORT

Chain E:

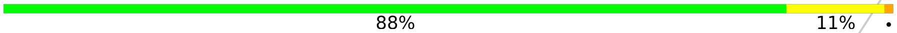

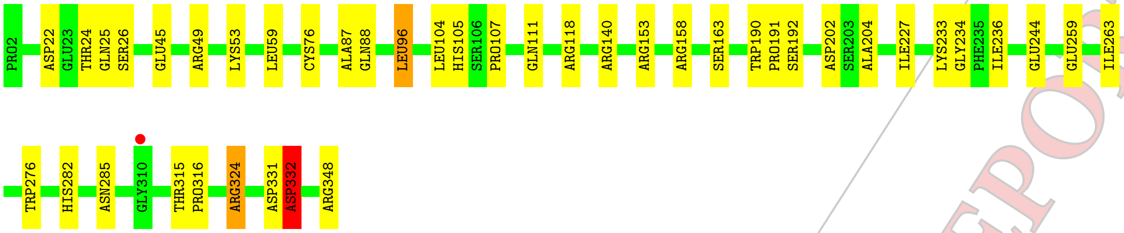

- Molecule 2:

Chain F:

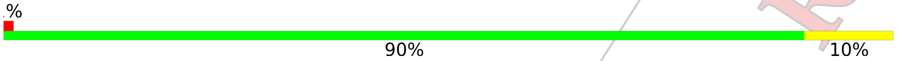

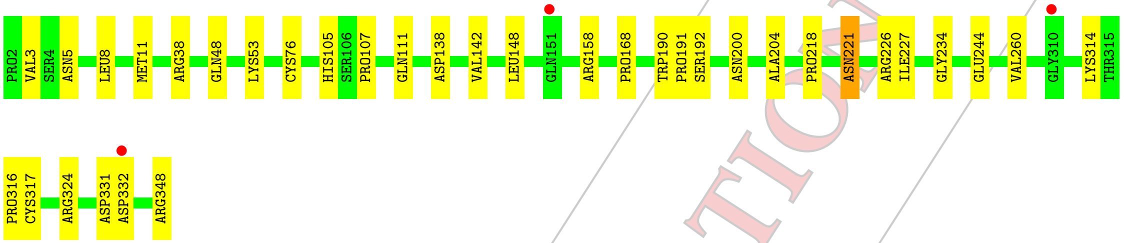

PRELIMINARY VALIDATION REPORT

4 Data and refinement statistics (i)

Property	Value	Source
Space group	P 21 21 2	Depositor
Cell constants<br>a, b, c, α, β, γ	125.96Å 143.75Å 118.69Å<br>90.00° 90.00° 90.00°	Depositor
Resolution (Å)	47.41 – 2.00<br>47.37 – 2.00	Depositor<br>EDS
% Data completeness<br>(in resolution range)	96.7 (47.41-2.00)<br>96.7 (47.37-2.00)	Depositor<br>EDS
R_{merge}	(Not available)	Depositor
R_{sym}	(Not available)	Depositor
$< I/\sigma(I) >$ [1]	3.14 (at 2.00Å)	Xtriage
Refinement program	REFMAC 5.8.0258	Depositor
R, R_{free}	0.171 , 0.213<br>0.182 , 0.222	Depositor<br>DCC
R_{free} test set	2013 reflections (1.43%)	wwPDB-VP
Wilson B-factor (Å^2)	25.9	Xtriage
Anisotropy	0.039	Xtriage
Bulk solvent k_{sol}(e/Å^3), B_{sol}(Å^2)	0.36 , 45.4	EDS
L-test for twinning[2]	$< \|L\| > = 0.50$, $< L^2 > = 0.33$	Xtriage
Estimated twinning fraction	No twinning to report.	Xtriage
F_o,F_c correlation	0.97	EDS
Total number of atoms	17845	wwPDB-VP
Average B, all atoms (Å^2)	30.0	wwPDB-VP

Xtriage's analysis on translational NCS is as follows: *The analyses of the Patterson function reveals a significant off-origin peak that is 43.38 % of the origin peak, indicating pseudo-translational symmetry. The chance of finding a peak of this or larger height randomly in a structure without pseudo-translational symmetry is equal to 1.7777e-04. The detected translational NCS is most likely also responsible for the elevated intensity ratio.*

[1] Intensities estimated from amplitudes.
[2] Theoretical values of $< |L| >$, $< L^2 >$ for acentric reflections are 0.5, 0.333 respectively for untwinned datasets, and 0.375, 0.2 for perfectly twinned datasets.

PRELIMINARY VALIDATION REPORT

5 Model quality (i)

5.1 Standard geometry (i)

Bond lengths and bond angles in the following residue types are not validated in this section: FE

The Z score for a bond length (or angle) is the number of standard deviations the observed value is removed from the expected value. A bond length (or angle) with $|Z| > 5$ is considered an outlier worth inspection. RMSZ is the root-mean-square of all Z scores of the bond lengths (or angles).

Mol	Chain	Bond lengths		Bond angles	
		RMSZ	#$\|Z\|$ >5	RMSZ	#$\|Z\|$ >5
1	A	0.70	0/2788	0.86	2/3788 (0.1%)
1	B	0.74	0/2788	0.89	4/3788 (0.1%)
1	C	0.75	1/2788 (0.0%)	0.90	0/3788
1	D	0.75	0/2788	0.88	1/3788 (0.0%)
2	E	0.72	1/2783 (0.0%)	0.89	3/3781 (0.1%)
2	F	0.71	0/2783	0.87	2/3781 (0.1%)
All	All	0.73	2/16718 (0.0%)	0.88	12/22714 (0.1%)

Chiral center outliers are detected by calculating the chiral volume of a chiral center and verifying if the center is modelled as a planar moiety or with the opposite hand.A planarity outlier is detected by checking planarity of atoms in a peptide group, atoms in a mainchain group or atoms of a sidechain that are expected to be planar.

Mol	Chain	#Chirality outliers	#Planarity outliers
1	C	0	1

All (2) bond length outliers are listed below:

Mol	Chain	Res	Type	Atoms	Z	Observed(Å)	Ideal(Å)
1	C	127	GLU	CD-OE1	5.39	1.31	1.25
2	E	26	SER	CA-CB	-5.05	1.45	1.52

All (12) bond angle outliers are listed below:

Mol	Chain	Res	Type	Atoms	Z	Observed(^{o})	Ideal(^{o})
1	B	324	ARG	NE-CZ-NH2	8.46	124.53	120.30
1	B	324	ARG	NE-CZ-NH1	-7.23	116.69	120.30
1	A	324	ARG	NE-CZ-NH2	-6.36	117.12	120.30
1	B	332	ASP	CB-CA-C	-6.33	97.73	110.40
2	E	324	ARG	NE-CZ-NH1	6.05	123.33	120.30

Continued on next page...

PRELIMINARY VALIDATION REPORT

Continued from previous page...

Mol	Chain	Res	Type	Atoms	Z	Observed(^{o})	Ideal(^{o})
1	A	324	ARG	NE-CZ-NH1	5.87	123.23	120.30
2	E	105	HIS	CB-CA-C	5.76	121.92	110.40
2	E	332	ASP	CB-CA-C	-5.45	99.51	110.40
1	D	38	ARG	NE-CZ-NH1	5.44	123.02	120.30
2	F	324	ARG	NE-CZ-NH1	5.31	122.96	120.30
1	B	331	ASP	CB-CA-C	-5.07	100.26	110.40
2	F	105	HIS	CB-CA-C	5.04	120.48	110.40

There are no chirality outliers.

All (1) planarity outliers are listed below:

Mol	Chain	Res	Type	Group
1	C	332	ASP	Peptide

5.2 Too-close contacts (i)

In the following table, the Non-H and H(model) columns list the number of non-hydrogen atoms and hydrogen atoms in the chain respectively. The H(added) column lists the number of hydrogen atoms added and optimized by MolProbity. The Clashes column lists the number of clashes within the asymmetric unit, whereas Symm-Clashes lists symmetry related clashes.

Mol	Chain	Non-H	H(model)	H(added)	Clashes	Symm-Clashes
1	A	2723	0	2653	34	0
1	B	2723	0	2653	27	0
1	C	2723	0	2653	29	0
1	D	2723	0	2653	22	0
2	E	2718	0	2648	27	0
2	F	2718	0	2648	23	0
3	G	6	0	0	0	0
4	S	1511	0	0	41	0
All	All	17845	0	15908	157	0

The all-atom clashscore is defined as the number of clashes found per 1000 atoms (including hydrogen atoms). The all-atom clashscore for this structure is 5.

All (157) close contacts within the same asymmetric unit are listed below, sorted by their clash magnitude.

Atom-1	Atom-2	Interatomic distance (Å)	Clash overlap (Å)
1:C:289:CYS:HB2	4:S:1492:HOH:O	1.34	1.26

Continued on next page...

PRELIMINARY VALIDATION REPORT

Continued from previous page...

Atom-1	Atom-2	Interatomic distance (Å)	Clash overlap (Å)
2:F:348:ARG:C	4:S:1217:HOH:O	1.96	1.03
2:E:348:ARG:C	4:S:1473:HOH:O	1.95	1.02
2:F:8:LEU:HA	2:F:11:MET:HE3	1.45	0.97
1:B:241:GLY:O	4:S:1476:HOH:O	1.85	0.93
1:D:107:PRO:O	1:D:111:GLN:HG2	1.68	0.92
2:E:22:ASP:OD1	2:E:24:THR:HG22	1.69	0.91
1:A:227:ILE:HD11	1:A:244:GLU:HB3	1.54	0.87
1:B:242:GLY:N	4:S:1474:HOH:O	1.84	0.86
1:C:13:GLU:OE2	2:E:49:ARG:NH2	2.08	0.86
1:B:227:ILE:HD11	1:B:244:GLU:HB3	1.56	0.85
2:F:221:ASN:ND2	4:S:1266:HOH:O	2.12	0.83
1:D:227:ILE:HD11	1:D:244:GLU:HB3	1.66	0.77
1:C:349:ALA:O	4:S:1340:HOH:O	2.02	0.77
1:D:186:ARG:HB3	4:S:1477:HOH:O	1.84	0.76
2:F:8:LEU:HA	2:F:11:MET:CE	2.19	0.73
1:D:186:ARG:HG3	4:S:1170:HOH:O	1.88	0.73
2:E:331:ASP:O	2:E:332:ASP:HB2	1.86	0.72
1:A:153:ARG:NH1	4:S:1234:HOH:O	2.22	0.72
1:C:225:GLU:OE1	4:S:1319:HOH:O	2.10	0.69
1:D:171:GLN:OE1	4:S:1430:HOH:O	2.11	0.68
1:B:148:LEU:HD22	1:B:344:PRO:HD3	1.75	0.68
2:E:331:ASP:O	2:E:332:ASP:CB	2.42	0.68
2:E:227:ILE:HD11	2:E:244:GLU:HB3	1.75	0.67
1:A:246:GLU:OE1	1:C:324:ARG:NH1	2.20	0.66
1:C:76:CYS:HA	1:C:104:LEU:HD13	1.77	0.65
1:D:76:CYS:HA	1:D:104:LEU:HD13	1.77	0.65
1:B:240:HIS:HD2	4:S:1325:HOH:O	1.79	0.65
1:B:225:GLU:HG3	1:B:244:GLU:OE2	1.98	0.64
1:D:92:GLU:OE2	4:S:1358:HOH:O	2.15	0.64
2:F:227:ILE:HD11	2:F:244:GLU:HB3	1.81	0.62
1:C:226:ARG:NH2	4:S:471:HOH:O	2.27	0.62
1:D:156:HIS:HE1	4:S:1495:HOH:O	1.82	0.61
1:C:349:ALA:C	4:S:1340:HOH:O	2.37	0.61
1:B:189:HIS:HE1	4:S:1468:HOH:O	1.82	0.61
2:E:25:GLN:NE2	2:E:118:ARG:HH12	1.99	0.61
1:A:173:PRO:O	1:A:197:THR:HG23	2.02	0.60
2:E:140:ARG:HD2	4:S:1224:HOH:O	2.02	0.60
1:A:204:ALA:HB3	1:A:234:GLY:HA2	1.84	0.58
1:C:102:MET:CE	1:C:288:MET:CE	2.81	0.58
2:E:88:GLN:HE22	2:E:111:GLN:HE21	1.49	0.58
1:B:331:ASP:O	1:B:332:ASP:CB	2.52	0.58

Continued on next page...

PRELIMINARY VALIDATION REPORT

Continued from previous page...

Atom-1	Atom-2	Interatomic distance (Å)	Clash overlap (Å)
2:F:158:ARG:NH1	4:S:1096:HOH:O	2.17	0.57
2:E:282:HIS:HE1	4:S:1470:HOH:O	1.87	0.57
1:A:49:ARG:NH2	1:B:13:GLU:OE1	2.32	0.56
2:F:3:VAL:HG11	2:F:11:MET:HE1	1.87	0.56
1:C:20:ARG:NH1	4:S:1191:HOH:O	2.39	0.56
1:A:13:GLU:OE2	1:B:49:ARG:NH2	2.39	0.55
2:E:107:PRO:O	2:E:111:GLN:HG3	2.07	0.55
2:F:3:VAL:HG11	2:F:11:MET:CE	2.36	0.55
2:F:48:GLN:NE2	4:S:375:HOH:O	2.34	0.55
1:C:101:MET:O	1:C:102:MET:HB3	2.06	0.55
1:D:107:PRO:O	1:D:111:GLN:CG	2.49	0.54
1:B:189:HIS:HD2	4:S:1469:HOH:O	1.90	0.54
1:B:331:ASP:O	1:B:332:ASP:HB2	2.08	0.54
2:E:76:CYS:O	2:E:316:PRO:HA	2.08	0.54
1:D:156:HIS:HB2	4:S:1491:HOH:O	2.07	0.54
1:B:241:GLY:CA	4:S:1474:HOH:O	2.58	0.52
1:D:101:MET:O	1:D:102:MET:HB3	2.09	0.52
1:A:49:ARG:CZ	1:B:49:ARG:HD2	2.40	0.52
1:A:47:ALA:HB3	1:A:54:VAL:HG11	1.93	0.50
1:D:260:VAL:HG12	1:D:317:CYS:HB3	1.92	0.50
1:D:166:HIS:HB2	4:S:1495:HOH:O	2.11	0.50
1:B:276:TRP:CE2	1:B:324:ARG:HD2	2.45	0.50
1:A:64:HIS:HD2	4:S:1475:HOH:O	1.94	0.50
1:C:349:ALA:C	4:S:1267:HOH:O	2.48	0.50
1:A:148:LEU:HD12	1:A:344:PRO:HD3	1.93	0.50
2:E:236:ILE:HD11	2:E:263:ILE:HD11	1.94	0.49
2:E:158:ARG:HA	2:E:163:SER:O	2.12	0.49
1:A:224:ARG:HD3	4:S:1162:HOH:O	2.13	0.49
1:D:276:TRP:CE2	1:D:324:ARG:HD3	2.48	0.48
1:C:102:MET:CE	1:C:288:MET:HE2	2.43	0.48
1:A:148:LEU:HD12	1:A:344:PRO:HG3	1.95	0.48
2:E:45:GLU:HB2	4:S:1363:HOH:O	2.13	0.48
1:B:102:MET:SD	1:B:288:MET:HE1	2.54	0.48
2:F:8:LEU:CA	2:F:11:MET:HE3	2.29	0.48
1:A:151:GLN:C	1:A:151:GLN:OE1	2.51	0.48
1:B:158:ARG:HA	1:B:163:SER:O	2.14	0.47
1:C:276:TRP:CD2	1:C:324:ARG:HD3	2.49	0.47
1:C:102:MET:CE	1:C:288:MET:HE1	2.44	0.47
1:C:243:PHE:N	4:S:1319:HOH:O	2.47	0.47
1:A:328:ILE:HD12	1:A:336:VAL:HG21	1.95	0.47
1:A:158:ARG:HA	1:A:163:SER:O	2.14	0.47

Continued on next page...

PRELIMINARY VALIDATION REPORT

Continued from previous page...

Atom-1	Atom-2	Interatomic distance (Å)	Clash overlap (Å)
1:B:106:SER:HB2	1:B:107:PRO:HD2	1.97	0.47
1:A:253:LYS:C	1:A:253:LYS:HD2	2.36	0.47
1:C:79:THR:HG22	1:C:104:LEU:HD12	1.96	0.46
1:A:276:TRP:CE2	1:A:324:ARG:HD2	2.50	0.46
2:E:96:LEU:HD12	2:E:118:ARG:HG3	1.96	0.46
2:E:285:ASN:HB2	4:S:1094:HOH:O	2.14	0.46
1:A:201:GLU:CG	4:S:869:HOH:O	2.63	0.46
1:C:72:MET:HB3	1:C:102:MET:HG2	1.98	0.46
1:C:102:MET:HE2	1:C:288:MET:CE	2.44	0.46
1:A:197:THR:O	1:A:265:HIS:HA	2.16	0.45
1:B:260:VAL:HG12	1:B:317:CYS:HB3	1.98	0.45
1:C:72:MET:CB	1:C:102:MET:HG2	2.46	0.45
1:C:276:TRP:CE2	1:C:324:ARG:HD3	2.51	0.45
2:E:25:GLN:HE22	2:E:118:ARG:HH12	1.64	0.45
1:A:307:THR:HG23	1:A:313:ARG:O	2.17	0.45
1:D:156:HIS:CE1	4:S:1495:HOH:O	2.63	0.45
1:B:342:VAL:HA	1:B:348:ARG:HH11	1.81	0.45
1:A:231:ILE:HA	1:A:235:PHE:O	2.17	0.44
2:E:236:ILE:CD1	2:E:263:ILE:HD11	2.47	0.44
2:F:168:PRO:HB2	2:F:200:ASN:ND2	2.32	0.44
1:A:148:LEU:HD12	1:A:344:PRO:CD	2.47	0.44
1:D:146:GLU:HG2	1:D:150:LYS:HE2	1.99	0.44
1:A:251:TYR:O	1:A:254:TYR:HB3	2.17	0.44
1:D:276:TRP:CD1	1:D:324:ARG:HD3	2.52	0.44
1:D:276:TRP:CD2	1:D:324:ARG:HD3	2.53	0.44
2:F:76:CYS:O	2:F:316:PRO:HA	2.17	0.44
1:C:107:PRO:O	1:C:111:GLN:OE1	2.36	0.44
2:F:107:PRO:O	2:F:111:GLN:HG3	2.17	0.44
2:F:168:PRO:HB2	2:F:200:ASN:HD22	1.83	0.44
1:A:173:PRO:O	1:A:197:THR:CG2	2.66	0.43
1:B:167:ALA:HB1	1:B:199:PRO:HB3	2.00	0.43
2:E:202:ASP:O	2:E:233:LYS:HA	2.18	0.43
1:B:224:ARG:HD3	4:S:1119:HOH:O	2.17	0.43
2:F:5:ASN:OD1	2:F:38:ARG:HD2	2.18	0.43
1:A:340:ASP:OD1	1:A:341:VAL:N	2.52	0.43
2:E:158:ARG:HB3	2:E:158:ARG:CZ	2.49	0.43
2:E:276:TRP:CE2	2:E:324:ARG:HD2	2.53	0.43
1:A:276:TRP:NE1	1:A:324:ARG:HD2	2.34	0.43
1:A:31:LYS:O	1:A:58:GLU:HA	2.19	0.43
2:F:204:ALA:HB3	2:F:234:GLY:HA2	2.01	0.43
2:F:148:LEU:HD12	2:F:148:LEU:HA	1.90	0.43

Continued on next page...

PRELIMINARY VALIDATION REPORT

Continued from previous page...

Atom-1	Atom-2	Interatomic distance (Å)	Clash overlap (Å)
2:E:76:CYS:HA	2:E:104:LEU:HD13	2.01	0.42
1:D:276:TRP:CG	1:D:324:ARG:HD3	2.55	0.42
1:B:148:LEU:HD13	1:B:344:PRO:HG3	2.01	0.42
1:D:260:VAL:CG1	1:D:317:CYS:HB3	2.49	0.42
1:C:102:MET:HE2	1:C:288:MET:HE1	2.02	0.42
1:B:230:GLU:OE1	1:B:240:HIS:HE1	2.03	0.42
1:D:102:MET:O	1:D:102:MET:HG3	2.20	0.42
2:F:226:ARG:NE	4:S:1386:HOH:O	2.52	0.42
1:C:102:MET:O	1:C:102:MET:HG3	2.20	0.41
1:C:125:PRO:HA	1:C:126:PRO:HD3	1.96	0.41
1:C:146:GLU:OE2	4:S:575:HOH:O	2.21	0.41
2:E:59:LEU:HD21	2:E:87:ALA:HB1	2.02	0.41
2:F:138:ASP:O	2:F:142:VAL:HG23	2.20	0.41
1:B:143:LEU:O	1:B:147:THR:HG23	2.20	0.41
2:F:260:VAL:HG12	2:F:317:CYS:HB3	2.00	0.41
1:A:208:LEU:HD12	1:A:231:ILE:HD11	2.02	0.41
1:A:133:LEU:HA	1:A:134:PRO:HD3	1.95	0.41
1:A:221:ASN:HD21	1:A:284:ARG:HH21	1.68	0.41
1:B:190:TRP:HA	1:B:191:PRO:HA	1.87	0.41
1:B:345:GLU:HA	1:B:348:ARG:HD3	2.03	0.41
2:F:331:ASP:O	2:F:332:ASP:CG	2.59	0.41
1:A:201:GLU:HG2	4:S:869:HOH:O	2.21	0.41
2:E:190:TRP:HA	2:E:191:PRO:HA	1.88	0.41
2:E:204:ALA:HB3	2:E:234:GLY:HA2	2.03	0.41
1:A:276:TRP:CD1	1:A:324:ARG:HD2	2.56	0.41
1:D:64:HIS:HE1	4:S:1479:HOH:O	2.02	0.41
2:F:190:TRP:HA	2:F:191:PRO:HA	1.89	0.40
1:A:260:VAL:HG12	1:A:317:CYS:HB3	2.03	0.40
1:C:20:ARG:HG2	4:S:1191:HOH:O	2.21	0.40
1:C:331:ASP:O	1:C:332:ASP:OD1	2.40	0.40
2:E:259:GLU:HB2	2:E:315:THR:HB	2.04	0.40
1:C:151:GLN:HE21	1:C:151:GLN:HB2	1.68	0.40
2:F:3:VAL:CG1	2:F:11:MET:HE2	2.51	0.40

There are no symmetry-related clashes.

PRELIMINARY VALIDATION REPORT

5.3 Torsion angles (i)

5.3.1 Protein backbone (i)

In the following table, the Percentiles column shows the percent Ramachandran outliers of the chain as a percentile score with respect to all X-ray entries followed by that with respect to entries of similar resolution.

The Analysed column shows the number of residues for which the backbone conformation was analysed, and the total number of residues.

Mol	Chain	Analysed	Favoured	Allowed	Outliers	Percentiles	
1	A	346/348 (99%)	331 (96%)	14 (4%)	1 (0%)	41	37
1	B	346/348 (99%)	332 (96%)	13 (4%)	1 (0%)	41	37
1	C	346/348 (99%)	333 (96%)	11 (3%)	2 (1%)	25	19
1	D	346/348 (99%)	330 (95%)	14 (4%)	2 (1%)	25	19
2	E	345/347 (99%)	330 (96%)	13 (4%)	2 (1%)	25	19
2	F	345/347 (99%)	332 (96%)	12 (4%)	1 (0%)	41	37
All	All	2074/2086 (99%)	1988 (96%)	77 (4%)	9 (0%)	34	30

All (9) Ramachandran outliers are listed below:

Mol	Chain	Res	Type
2	E	332	ASP
1	B	192	SER
1	D	192	SER
1	A	192	SER
1	C	102	MET
1	C	192	SER
2	E	192	SER
1	D	102	MET
2	F	192	SER

5.3.2 Protein sidechains (i)

In the following table, the Percentiles column shows the percent sidechain outliers of the chain as a percentile score with respect to all X-ray entries followed by that with respect to entries of similar resolution.

The Analysed column shows the number of residues for which the sidechain conformation was analysed, and the total number of residues.

PRELIMINARY VALIDATION REPORT

Mol	Chain	Analysed	Rotameric	Outliers	Percentiles	
1	A	287/287 (100%)	280 (98%)	7 (2%)	49	51
1	B	287/287 (100%)	277 (96%)	10 (4%)	36	35
1	C	287/287 (100%)	282 (98%)	5 (2%)	60	65
1	D	287/287 (100%)	282 (98%)	5 (2%)	60	65
2	E	287/287 (100%)	284 (99%)	3 (1%)	76	81
2	F	287/287 (100%)	283 (99%)	4 (1%)	67	72
All	All	1722/1722 (100%)	1688 (98%)	34 (2%)	55	58

All (34) residues with a non-rotameric sidechain are listed below:

Mol	Chain	Res	Type
1	A	105	HIS
1	A	137	ASP
1	A	151	GLN
1	A	197	THR
1	A	225	GLU
1	A	253	LYS
1	A	314	LYS
1	B	49	ARG
1	B	96	LEU
1	B	104	LEU
1	B	105	HIS
1	B	153	ARG
1	B	169	LEU
1	B	189	HIS
1	B	191	PRO
1	B	225	GLU
1	B	333	LYS
1	C	53	LYS
1	C	85	LEU
1	C	114	LYS
1	C	160	LYS
1	C	307	THR
1	D	17	LYS
1	D	53	LYS
1	D	106	SER
1	D	160	LYS
1	D	307	THR
2	E	53	LYS
2	E	96	LEU
2	E	153	ARG

Continued on next page...

PRELIMINARY VALIDATION REPORT

Continued from previous page...

Mol	Chain	Res	Type
2	F	53	LYS
2	F	218	PRO
2	F	221	ASN
2	F	314	LYS

Some sidechains can be flipped to improve hydrogen bonding and reduce clashes. All (14) such sidechains are listed below:

Mol	Chain	Res	Type
1	A	64	HIS
1	A	221	ASN
1	A	240	HIS
1	A	256	ASN
1	B	189	HIS
1	B	240	HIS
1	C	48	GLN
1	C	151	GLN
1	D	109	GLN
1	D	221	ASN
2	E	25	GLN
2	E	111	GLN
2	E	282	HIS
2	F	221	ASN

5.3.3 RNA (i)

There are no RNA molecules in this entry.

5.4 Non-standard residues in protein, DNA, RNA chains (i)

There are no non-standard protein/DNA/RNA residues in this entry.

5.5 Carbohydrates (i)

There are no monosaccharides in this entry.

5.6 Ligand geometry (i)

Of 6 ligands modelled in this entry, 6 are monoatomic - leaving 0 for Mogul analysis.

PRELIMINARY VALIDATION REPORT

There are no bond length outliers.

There are no bond angle outliers.

There are no chirality outliers.

There are no torsion outliers.

There are no ring outliers.

No monomer is involved in short contacts.

5.7 Other polymers (i)

There are no such residues in this entry.

5.8 Polymer linkage issues (i)

There are no chain breaks in this entry.

PRELIMINARY VALIDATION REPORT

6 Fit of model and data (i)

6.1 Protein, DNA and RNA chains (i)

In the following table, the column labelled '#RSRZ> 2' contains the number (and percentage) of RSRZ outliers, followed by percent RSRZ outliers for the chain as percentile scores relative to all X-ray entries and entries of similar resolution. The OWAB column contains the minimum, median, 95th percentile and maximum values of the occupancy-weighted average B-factor per residue. The column labelled 'Q< 0.9' lists the number of (and percentage) of residues with an average occupancy less than 0.9.

Mol	Chain	Analysed	<RSRZ>	#RSRZ>2			OWAB(Å^2)	Q<0.9
1	A	348/348 (100%)	-0.27	4 (1%)	80	79	18, 30, 50, 69	0
1	B	348/348 (100%)	-0.39	0	100	100	17, 26, 44, 60	0
1	C	348/348 (100%)	-0.29	0	100	100	18, 26, 46, 82	0
1	D	348/348 (100%)	-0.29	0	100	100	16, 24, 42, 74	0
2	E	347/347 (100%)	-0.38	1 (0%)	94	93	17, 27, 47, 59	0
2	F	347/347 (100%)	-0.37	3 (0%)	84	83	17, 28, 47, 67	0
All	All	2086/2086 (100%)	-0.33	8 (0%)	92	92	16, 27, 47, 82	0

All (8) RSRZ outliers are listed below:

Mol	Chain	Res	Type	RSRZ
1	A	332	ASP	2.7
2	F	332	ASP	2.7
2	F	310	GLY	2.5
2	F	151	GLN	2.4
1	A	333	LYS	2.2
2	E	310	GLY	2.2
1	A	153	ARG	2.1
1	A	147	THR	2.1

6.2 Non-standard residues in protein, DNA, RNA chains (i)

There are no non-standard protein/DNA/RNA residues in this entry.

6.3 Carbohydrates (i)

There are no monosaccharides in this entry.

PRELIMINARY VALIDATION REPORT

6.4 Ligands (i)

In the following table, the Atoms column lists the number of modelled atoms in the group and the number defined in the chemical component dictionary. The B-factors column lists the minimum, median, 95th percentile and maximum values of B factors of atoms in the group. The column labelled 'Q< 0.9' lists the number of atoms with occupancy less than 0.9.

Mol	Type	Chain	Res	Atoms	RSCC	RSR	B-factors(Å^2)	Q<0.9
3	FE	G	1	1/?	0.80	0.08	43,43,43,43	0
3	FE	G	4	1/?	0.84	0.09	36,36,36,36	0
3	FE	G	6	1/?	0.85	0.11	44,44,44,44	0
3	FE	G	3	1/?	0.86	0.12	39,39,39,39	0
3	FE	G	2	1/?	0.88	0.11	38,38,38,38	0
3	FE	G	5	1/?	0.97	0.08	38,38,38,38	0

6.5 Other polymers (i)

There are no such residues in this entry.

PRELIMINARY VALIDATION REPORT
