# Supplementary figures and images for: Structure-guided insights into heterocyclic ring-cleavage catalysis of the non-heme Fe (II) dioxygenase NicX
Source: Nat Commun. 2021 Feb 26;12:1301. doi: 10.1038/s41467-021-21567-9 (PMC7910607; doi:10.1038/s41467-021-21567-9)

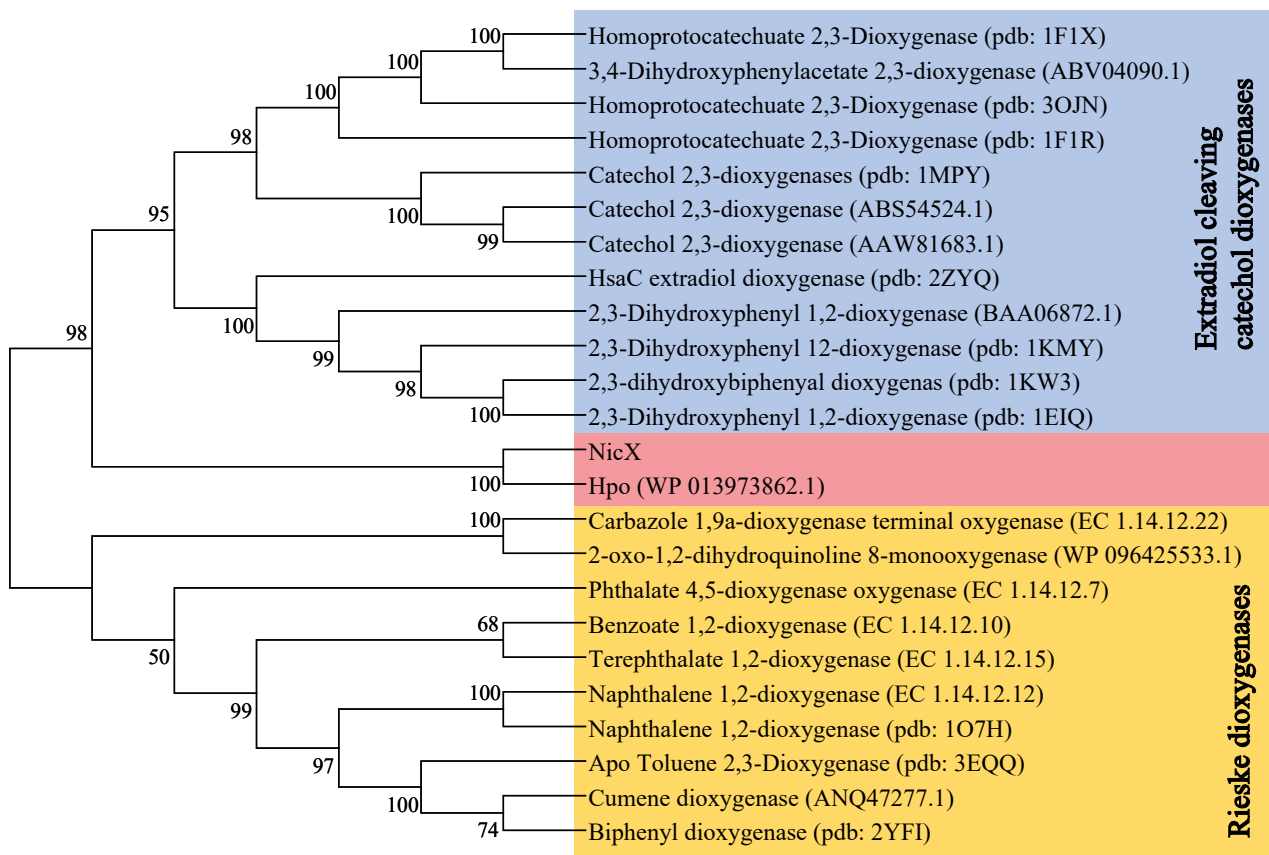

Supplement: Supplementary file 6 — Source Data [file 41467_2021_21567_MOESM6_ESM.zip › Source data/Source data 5-Raw Figures/Figure 1.pdf]

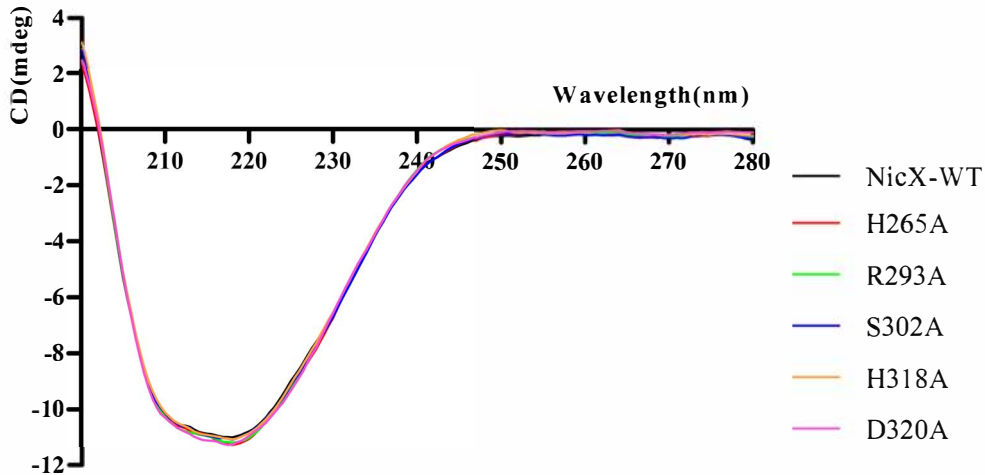

**Circular dichroism spectra of NicX and its mutants**

Supplement: Supplementary file 6 — Source Data [file 41467_2021_21567_MOESM6_ESM.zip › Source data/Source data 5-Raw Figures/Supplementary Figure 1.pdf]
